# An integrative approach to identify hexaploid wheat miRNAome associated with development and tolerance to abiotic stress

**DOI:** 10.1186/s12864-015-1490-8

**Published:** 2015-04-24

**Authors:** Zahra Agharbaoui, Mickael Leclercq, Mohamed Amine Remita, Mohamed A Badawi, Etienne Lord, Mario Houde, Jean Danyluk, Abdoulaye Baniré Diallo, Fathey Sarhan

**Affiliations:** Department of Biological Sciences, University of Quebec in Montreal, Montreal, Canada; Department of Computer Sciences, University of Quebec in Montreal, Montreal, Canada; School of Computer Science and McGill Centre for Bioinformatics, McGill University, Montreal, QC Canada

**Keywords:** Abiotic stress, Development, Deep sequencing, MiRNA prediction, Expressed sequenced tags, *Triticum aestivum. L*, Vernalization

## Abstract

**Background:**

Wheat is a major staple crop with broad adaptability to a wide range of environmental conditions. This adaptability involves several stress and developmentally responsive genes, in which microRNAs (miRNAs) have emerged as important regulatory factors. However, the currently used approaches to identify miRNAs in this polyploid complex system focus on conserved and highly expressed miRNAs avoiding regularly those that are often lineage-specific, condition-specific, or appeared recently in evolution. In addition, many environmental and biological factors affecting miRNA expression were not yet considered, resulting still in an incomplete repertoire of wheat miRNAs.

**Results:**

We developed a conservation-independent technique based on an integrative approach that combines machine learning, bioinformatic tools, biological insights of known miRNA expression profiles and universal criteria of plant miRNAs to identify miRNAs with more confidence. The developed pipeline can potentially identify novel wheat miRNAs that share features common to several species or that are species specific or clade specific. It allowed the discovery of 199 miRNA candidates associated with different abiotic stresses and development stages. We also highlight from the raw data 267 miRNAs conserved with 43 miRBase families. The predicted miRNAs are highly associated with abiotic stress responses, tolerance and development. GO enrichment analysis showed that they may play biological and physiological roles associated with cold, salt and aluminum (Al) through auxin signaling pathways, regulation of gene expression, ubiquitination, transport, carbohydrates, gibberellins, lipid, glutathione and *secondary metabolism,* photosynthesis, as well as floral transition and flowering.

**Conclusion:**

This approach provides a broad repertoire of hexaploid wheat miRNAs associated with abiotic stress responses, tolerance and development. These valuable resources of expressed wheat miRNAs will help in elucidating the regulatory mechanisms involved in freezing and Al responses and tolerance mechanisms as well as for development and flowering. In the long term, it may help in breeding stress tolerant plants.

**Electronic supplementary material:**

The online version of this article (doi:10.1186/s12864-015-1490-8) contains supplementary material, which is available to authorized users.

## Background

Abiotic stresses such as cold, drought, salt and aluminum (Al) limit plant growth and development, causing reduction in crop yield and important economic losses for farmers. To tolerate these stresses, plants have evolved a broad spectrum of metabolic, physiological and developmentally adaptations. These adaptive changes are under the control of dynamic networks of genetic regulatory mechanisms that involve a large number of stress responsive genes. MicroRNAs (miRNAs), a major class of small non-coding RNAs, have emerged as key regulators of gene expression at the post-transcriptional level during plant growth and development [1-3]. Several studies have shown that many miRNA families are involved in response to different abiotic stresses in many species [4-7]. A large number of plant miRNAs and their targets have been identified in the plant model *Arabidopsis thaliana* and many other species. Recent results have shown that plant miRNA genes are dispersed throughout the genome [8] within protein coding genes [8,9], introns of protein coding and non-coding genes, and in intergenic regions [10,11]. Moreover, miRNAs may be produced from repetitive transposable elements [12,13]. To date, at the best of our knowledge, 2707 wheat miRNA candidates were identified by both bioinformatics and experimental approaches, using wheat expressed sequence tags (EST) database, the available genomic sequences of the hexaploid wheat genome, its individual chromosome arms and its ancestors [6,13-29]. Among the wheat miRNA published sequences, 237 are registered in miRBase, a database of experimental miRNAs [30], and 170 are registered in PMRD, a database of plant miRNAs identified using an *in silico* approach [31]. Although the wheat genome is completely sequenced, it is not yet possible to perform a thorough genome-wide study in the hexaploid wheat *T. aestivum* since the genome is not completely assembled and annotated. This is caused by its large and complex genome containing a high percentage of DNA repeats (hexaploid genome AABBDD with approximately 1.7 × 10^10^ bp with at least 80% of DNA repeats) [32]. *In silico* approaches for the prediction of miRNAs include screening genomic or EST databases for orthologous sequences of known miRNAs and analyzing their pre-miRNA hairpin structures. Although these approaches were successful in identifying conserved miRNAs in plants that have their genomes fully sequenced and annotated [10,33,34], they eliminate the potential of searching for low abundance miRNAs that are often lineage-specific [35] or condition-specific [36] or that appeared recently in evolution (young miRNAs). The challenge is bigger using polyploid species with partially sequenced and assembled genome such as the hexaploid wheat having a high content of repetitive DNA. To tackle this issue, one should develop conservation-independent techniques based on structure analyses and/or expression pattern of dicer cleavage products among pre-miRNAs [37].

Most computational approaches labeled as miRNA predictors are actually pre-miRNA predictors, in the sense that they identify candidate genomic regions that may form pre-miRNAs but rarely take into account the availability of candidate mature miRNA evidence within the pre-miRNA. Several tools such as miRDeep [37,38], miRanalyzer [39,40] and MiRdup [41] were developed to predict miRNAs from raw reads data and shown to be accurate in most cases. Furthermore, many factors that affect miRNA expression including genotypes, tissues, age, development stage, growth condition (soil, hydroponic solution, temperature, humidity and photoperiod), stress treatment, are rarely considered in previous wheat miRNA identification studies. All wheat reported miRNAs were identified in libraries produced from seedlings or plants grown under normal conditions [14,21,23,26,31], or tissue exposed to heat [15] or seedling [28] and pollen mother cells from plants [6] exposed to cold stress [6], or drought [16]. They were identified from different genotypes of winter or spring wheat in soil, or hydroponic solution and under different photoperiod conditions, or in field conditions. Since miRNA expression is tissue specific and regulated in response to plant development and growth conditions, the miRNA repertoire of hexaploid wheat is still incomplete. Although a large number of miRNAs associated with development or some abiotic stresses in wheat were previously identified, their functional diversity in Al, freezing tolerance, and floral transition in winter wheat is still unknown. Hence, the identification of miRNAs associated with tolerance to abiotic stress and floral transition is a first step towards the elucidation of their role in wheat.

**Figure 1:**
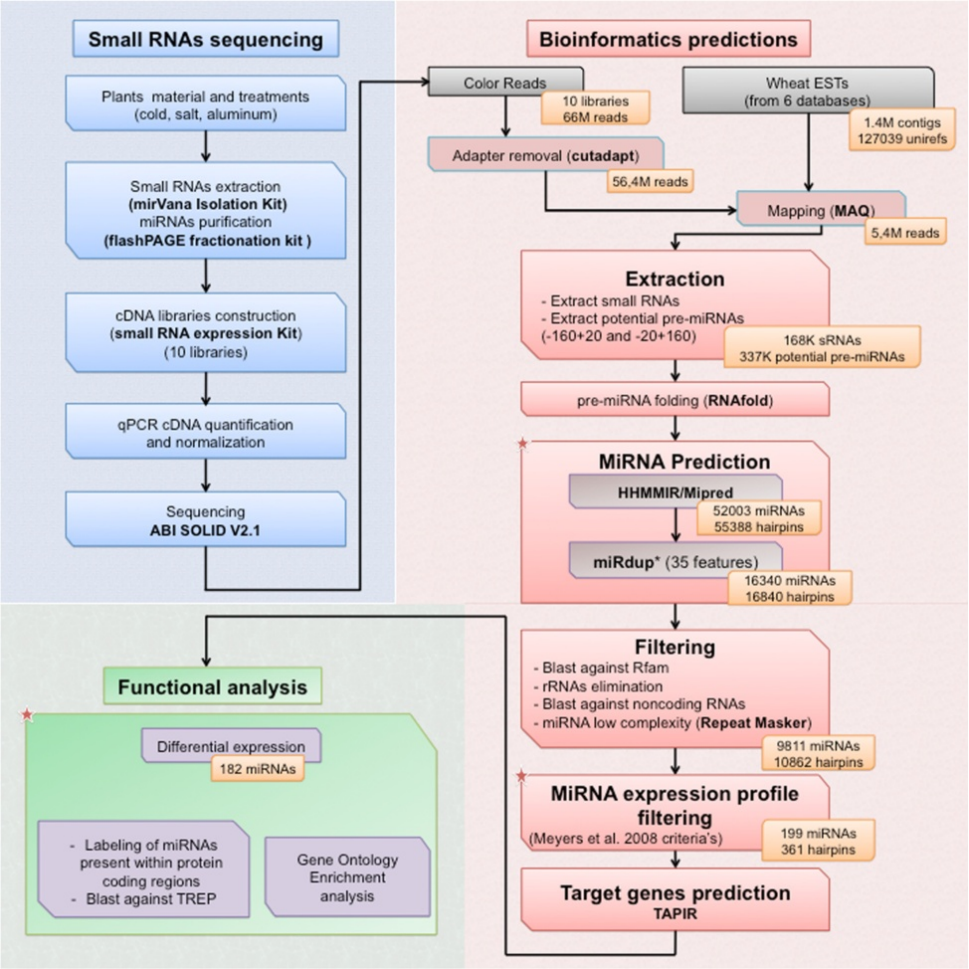
Overview of the wheat miRNA pipeline. The procedure is divided in three parts: producing and sequencing small RNA libraries, the bioinformatic prediction of miRNAs and functional analysis of the predicted miRNAs. The customized or developed steps are marked by stars. Orange boxes specify the data at hand after each given step. For details see Experimental procedure.

To ensure an accurate identification of a large fraction of miRNAs associated with different physiological conditions in both stress sensitive and tolerant wheat, we conducted the present study to: 1) identify miRNAs from different tissues of plants from different genotypes grown under different stress conditions (cold, salt and aluminum) and at different development stages (vegetative and reproductive phases); 2) develop an integrative pipeline that combines bioinformatic tools, biological insights about known miRNA expression and dicer ligation patterns according to the universal plant miRNA criteria [37,38], miRNA expression profiles in deep sequencing data [42], functional classification and experimental approaches (Figure 1). The bioinformatic tools include Mipred [43], HHMMiR [44], MirCheck [45] and MiRdup*, a plant updated version of our machine learning MiRdup [41] which validates the position of sequenced miRNAs in its corresponding folded pre-miRNA. Our integrative approach allows the discovery and profile of 165 novel hexaploid wheat abiotic stress responsive candidate miRNAs including ones associated with cold (52 miRNAs) and Al (27 miRNAs) tolerance as well as 99 developmentally responsive miRNAs with a high confidence level. It is the first study to report a large scale identification of hexaploid miRNome miRNAs from different tissues of sensitive and tolerant genotypes under normal conditions and short/long exposure to different abiotic stresses during vegetative and/or reproductive phase.

## Results

### Identification of miRNA candidates and their targets in hexaploid wheat

Our miRNA discovery pipeline consists of more than twenty steps divided in three main parts: producing and sequencing small RNAs, predicting miRNAs from deep sequencing data, classifying predicted miRNAs based on their expression profiles and Gene Ontology (GO) of their target genes (Figure 1). The sequencing of ten constructed libraries from three wheat genotypes grown under different abiotic stress conditions and development stages (Additional file 1: Method S1) yielded a total of 89,105,096 redundant raw reads (66,400,401 distinct reads) with 56% of high sequence quality (Additional file 2: Table S2). Before mapping the raw reads, we collected 1.4 million wheat ESTs from several databases, clustered into 127,039 Uniref clusters yielding to the best of our knowledge, the largest well-annotated EST databank in wheat (Additional file 1: Method S2). After raw reads adapter removal, a total of 56.4 million unique raw reads were mapped to our collected EST database resulting of 5.4 million unique mapped sequences (Figure 1). We identified 168,834 small RNAs and extracted 337,668 potential pre-miRNAs. Among the extracted pre-miRNA, 17,180 and 39,144 potential hairpins satisfy the minimal structural criteria of miRNAs assessed by two well-known pre-miRNA predictors, MiPred [43] and HHMMiR [44], respectively (Figure 1). The lack of consideration of the mature miRNA localization in the hairpin, the expression profiles of reads throughout the hairpin, and/or other evidence of plant-miRNA characteristics represent major causes of overestimation of the number of candidate hairpins. We used a two-step method of miRNA identification within its hairpin from the folded pre-miRNA using MiRdup* and expression profile filtering using Meyers *et al*. relaxed criteria [42] (Figure 1). In the first step, MiRdup* trained on experimentally validated miRNAs from different datasets (all miRBase species, all plants and monocots only) localizes miRNA positions in pre-miRNAs (Additional file 1: Method S4 and Additional file 2: Table S3). The use of MiRdup* reduced the number of pre-miRNA hairpin candidates by 81% (Additional file 3: Figures S1 and S2). In the second step, the expression profile filtering was based on miRNA expression pattern using the abundance of the miRNA candidates in each library and the distribution of reads mapped to a candidate pre-miRNA according to Meyers *et al*. relaxed criteria [42]. This allowed the elimination of the miRNA dicer-Like candidates and the reduction of miRNA candidates by 84%.

Overall, this method results in the identification of more candidates (Additional file 3: Figures S1 and S2) compared to Meyers *et al*., [42] and MIRcheck [45]. Consequently, it yields pre-miRNA candidates that have various ranges of secondary structures as shown in dotbracket notation (Additional file 4: data SD1). Taken together, our approach identifies 199 unique miRNA candidates associated with 361 pre-miRNAs (Additional file 4: data SD1). It is important to notice that the majority of reads (95%) and the predicted miRNAs (64%) have the highest quality value for their sequence (Additional file 2: Table S4). It is important to note that, MiRdup* captures 95% of the miRNAs identified using MIRcheck with the same criteria from Meyers *et al*. [42] while 151 putative candidates (containing validated candidates) are excluded by MIRcheck (Additional file 3: Figures S2). In addition, our pipeline identified miRNAs that have features that are species specific, clade specific or shared between several species. We found that among the 199 identified miRNAs, 147 were identified by MiRdup* trained on all species of miRBase; only 49 were commonly identified by MiRdup* trained on the three datasets (all miRBase, all plants, monocot only). This suggests that these miRNAs share common features with all the widely separated plant lineages recorded in the database miRBase. For instance, apMir_22246 corresponding to miR160 with perfect match in wheat and moss *Physcomitrella patens* (ppt-miR160) is highly expressed in our investigated conditions indicating that this miRNA may play common biological functions in plants kingdom. While 109 miRNAs were only identified by MiRdup* trained on all plant and 92 miRNAs when trained on only monocot.

The number of identified miRNAs may be an overestimation due to the redundancy created by similar but not identical ESTs in part due to the polyploid nature of wheat. In the latter scenario, two or more closely related ESTs (true homeologs or ESTs with SNP differences) could encode identical or closely related miRNAs. Furthermore expressed isomiRNAs that share the same properties with the real miRNA in one library could be the dominant functional in another library. The Additional file 3: Figure S3a highlights that the majority (about 69%) of the predicted miRNAs are associated with one pre-miRNA. Furthermore, most of the pre-miRNA candidates (93%) harbour a unique miRNA leading to an exclusive miRNA/pre-miRNA association (Additional file 3: Figure S3b). To characterize further the nature of the pre-miRNA candidates, we determined if they were associated with repetitive transposable elements and protein coding regions. The results revealed that 20% of pre-miRNAs that correspond to 6.5% of miRNA candidates overlap with transposable elements (at e-value of 5E-5 with 80% identity) from TREP database (Additional file 2: Table S5a). In addition, 15% of ESTs corresponding to less than 5% of miRNA candidates overlap partly with protein-coding regions (at e-value of 1E-20 with 75% identity) from protein plant database (Additional file 2: Table S5b).

Prediction of miRNA targets is an important step to elucidate miRNA function in regulating gene expression. Among the identified candidates, 67% (133/199) were predicted to have Uniref annotated target genes (Additional file 2: Table S6). Unlike animal target genes, it is generally accepted that plant targets adopt a perfect seed match with the corresponding miRNAs, allowing more accuracy in their prediction. We found that 37 miRNA candidates are predicted to target a unique gene identified as UniRef (Additional file 3: Figure S3c). Although the majority of miRNA candidates seem to have more than two targets, detailed analysis reveals that in many cases the targets annotated with different UniRefs have the same gene description (Additional file 2: Table S7). To better explore the functional properties of the target genes, GO analyses were performed (Additional file 3: Figure S3d).

We computed the enrichment of main GO Slim terms found within these targets based on the three GO categories (Additional file 3: Figures S4a-6a). Table 1 shows the enriched GO Slim terms and relevant associated target genes in libraries. An extensive description of GO enrichment analysis is presented in Additional file 5. Our results revealed that miRNA candidates target regulators, cell metabolism and transport genes. The regulatory genes are enriched for many transcription factors and protein families (Table 1 and Additional file 2: Table S7). They are involved in regulation of gene expression, signal transduction pathways and ubiquitin-mediated protein modifications (GO Slim *Nucleus* with P-value = 9.1e-004, GO Slim *DNA binding* with P-value from 1.0e-003 to 1.0e-005, GO Slim *DNA metabolic process* with P-value = 5.5e-004, GO Slim *protein binding* with P-value = 1.2e-006). Cellular metabolism genes are involved in hormone, lipid and carbohydrates metabolism (GO Slim *catalytic activity* with P-value = 3.4e-006), amino acid metabolism (GO Slim *secondary metabolic process* with P-value = 3.7e-008) (Additional file 2: Table S7).

**Table 1:** **Selected GO Slim enrichment in the different libraries and their relevant target genes**

	**L1**	**L2**	**L3**	**L4**	**L5**	**L6**	**L7**	**L8**	**L9**	**L10**	**All**	**GO Slim description**	**Relevant associated targets**
**CC**			**++**									Nucleus	CBFIVb-B20, MIKC-type MADS-box transcription factor, Auxin-responsive protein, Homeobox-leucine zipper protein, Histones, E3 ubiquitin-protein ligase
**M F**			**+**		**++**			**+**	**++**	**++**		DNA binding	DEAD-box ATP-dependent RNA helicase, Homeobox-leucine zipper proteins, Histones
					**+++**							Catalytic	Gibberellin 20 oxidase , Lipoxygenase, Glutathione peroxidase, Superoxide dismutase, Fructose-bisphosphate aldolase, Alcohol dehydrogenase, NADP-malic enzyme
				**++**			**++**					Transferase	Caffeic acid 3-O-methyltransferase, Tricetin 3',4',5'-O-trimethyltransferase, Serine/threonine-protein kinase, Glutathione-S-transferase
	**+++**	**+++**		**+++**		**+++**	**+++**				**+++**	Lipid binding	Non-specific lipid-transfer protein, Homeobox-leucine zipper proteins
	**++**	**++**			**++**			**+++**	**+++**	**++**	**+++**	Protein binding	Histones, WCOR719, DNA-directed RNA polymerases, MIKC-type MADS-box transcription factors, Serine/threonine-protein kinase, Cullin-1
**BP**	**+**	**+**										Transport	Sodium/hydrogen exchanger, Triose phosphate translocator, Protein transport protein, Non-specific lipid-transfer protein, Sucrose transporters
	**++**		**+++**	**++**	**+**	**+**	**++**	**++**	**++**	**++**		Resp. to endogenous stimulus	Auxin-responsive protein, Homeobox-leucine zipper protein, Myo-inositol 1-phosphate synthase
			**++**				**+**					Multicellular organismal dev.	Ubiquitin-like-specific protease, MIKC-type MADS-box transcription factors, Homeobox-leucine zipper protein
		**++**				**+**					**++**	Flower dev.	DL related protein, Ubiquitin-like-specific protease
									**++**			DNA metabolic process	Replication factor C, Histones, BARE-1 polyprotein
					**+++**						**++**	Sec. metabolic process	Phenylalanine ammonia-lyase, Tricetin 3',4',5'-O-trimethyltransferase

The enrichment is presented in four different symbols (“+++” for high (P-value < 10^−5^), “++” for medium (P-value < 10^−3^), “+” for low (P-value < 0.05) and “no symbol” for no enrichment (P-value ≥ 0.05). CC, cell component, MF, molecular function, BP, biological process. For details about the libraries and investigated conditions see Additional file 1 and Method S1; and about GO Slim terms classification and associated target genes, see Additional file 3: Figures S4a-6a.

### Characteristics of the miRNA candidates

**Figure 2:**
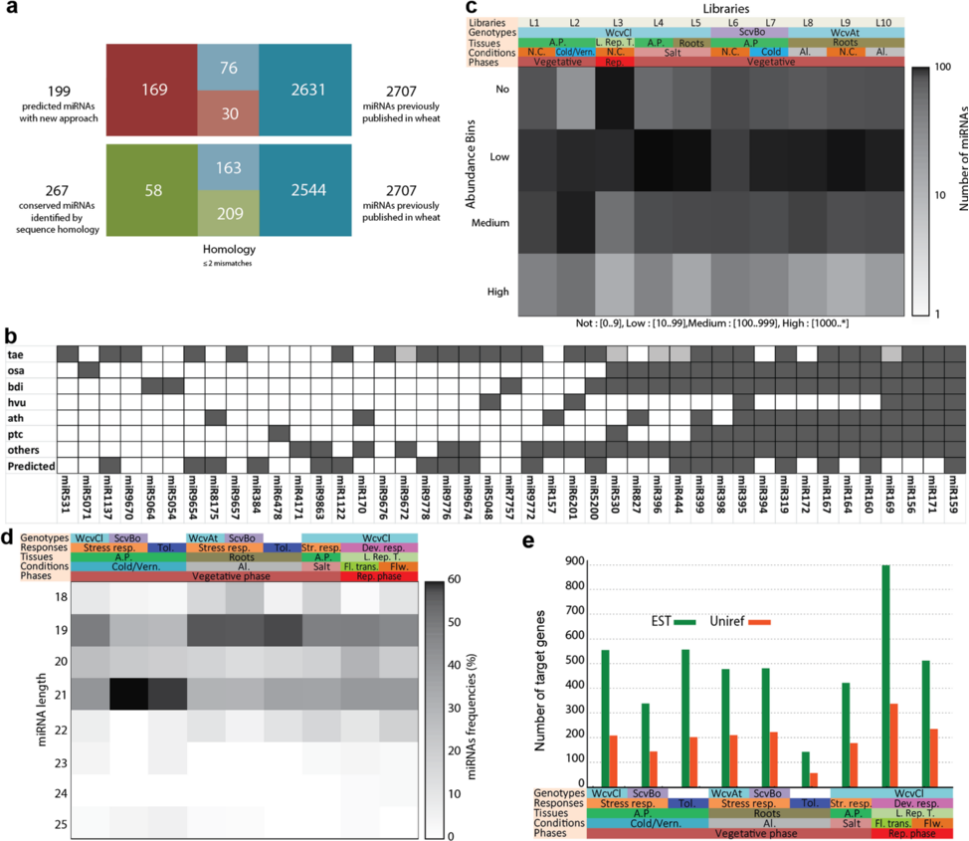
Overview of the predicted miRNAs. **a)** Diagram of the intersection between miRNAs predicted by the novel approach, conserved miRNAs identified by sequence homology, and miRNAs published in the literature; **b)** Evidence of conserved plant miRNA families present in miRBase including those predicted by our approach (tae, osa, bdi, hvu, ath and ptc correspond respectively to *Triticum aestivum, Oryza sativa, Brachypodium distachyon, Hordeum vulgare L., Arabidopsis thaliana and Populus trichocarpa*); **c)** The abundance bins of all predicted miRNAs in the 10 libraries (L1-L10) produced from plants grown under different investigated conditions. The abundance of the identified miRNAs represents the number of reads sequenced in each library and classified on 4 levels: low, 10–99 reads; medium, 100–999 reads; and high, 1000 and up; **d)** the length distribution of miRNAs associated with miRNAs differentially expressed in different investigated comparisons; **e)** the number of miRNA targeted genes (presented by EST id or UniRef id) associated with miRNAs differentially expressed in different investigated comparisons. WcvCl, winter wheat cultivar Clair (cold tolerant); ScvBo, spring wheat cultivar Bounty (cold and Al sensitive); WcvAt, winter wheat cultivar Atlas (Al tolerant); A.P, aerial parts; L./Rep. T., leaves and reproductive tissues; N.C, normal conditions; Al, Aluminum; Vern., vernalization; Rep., reproductive; Str. Resp., stress response; Tol., tolerance; Dev. resp., developmentally response; Fl. Trans, floral transition; Flw., flowering. See Additional file 1: Method S1 and Additional file 2: Table S1 for libraries and conditions and Additional file 2: Table S10 for the different investigated comparisons.

Among the 199 predicted miRNAs, 30 have sequence homology with 76 published miRNA in wheat (Figure 2a). In addition, we explored the potential of conserved miRBase families present in our raw data that could not be mapped into the EST and found 267 miRNAs, corresponding to 43 families from which 25 families are known in wheat [13-16,18-23,31,46-48] and 18 families have homology with many plant species (Figure 2b). It is important to notice that 13 families (58 miRNAs) have not yet been reported in previous wheat miRNA identification studies recorded in miRBase (Figure 2a). One can notice that the expression patterns of predicted miRNAs are different between the 10 libraries (Figure 2c). The abundance of 160 miRNAs corresponds at medium expression (abundance between 100–999 reads) in at least one library and 39 miRNAs have high reads abundance. MiRNAs were also classified according to their expression proportion over the total reads mapping to the corresponding pre-miRNAs [49]. Hence, one class would correspond to *typical miRNA* when its expression represents more than 50% of the expressed small RNAs mapping a given pre-miRNA (Additional file 2: Table S8 and Additional file 4: data SD2) [50]. Above 80% of the predicted miRNAs in each library correspond to typical miRNAs (Additional file 2: Table S8) and they correspond to highly confident expressed miRNAs (Additional file 4: data SD2).

The diversity of predicted miRNA sequences is greater at 19 nt in length in all libraries (Additional file 3: Figure S7a-d) while the diversity of conserved miRNAs is greater at 21 nt (Additional file 3: Figure S7e) (unique sequences). For redundant miRNA sequences, a major peak at 21 nt was observed for both predicted and conserved miRNAs (Additional file 3: Figure S7a-e). In addition, the majority of the identified miRNA expressed in the different investigated conditions are 19 or 21 nt long depending on the tissues, stresses, growth conditions or genotypes (Figure 2d). These miRNAs were shown to regulate at least 150 targets (60 unirefs) and at most 900 targets (335 unirefs) in all the explored conditions (Figure 2e).

### Confirmation of predicted miRNA candidates

**Figure 3:**
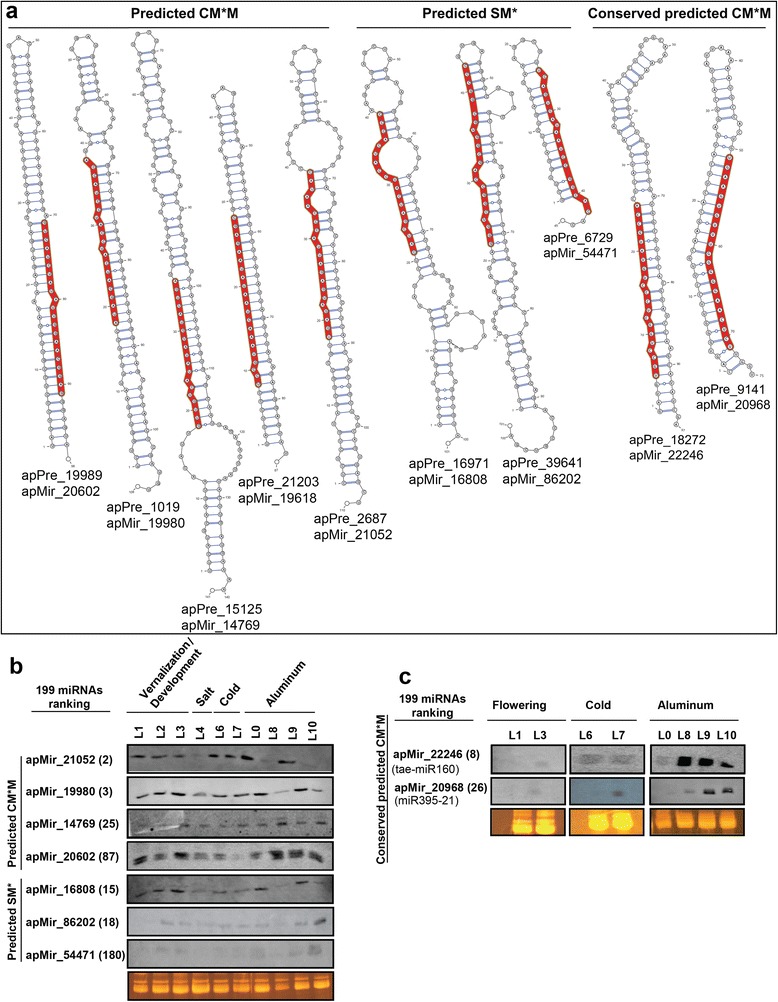
Experimental validation of predicted and conserved wheat miRNAs. **a)** Pre-miRNA secondary structure of miRNA candidates experimentally validated by northern in the investigated libraries; **b)** northern blot of predicted miRNAs in common between MiRdup* and MIRcheck (CM*M) as well as specifically predicted with MiRdup* tool (SM*); **c)** northern blot of miRNA candidates identified by both sequence homology against miRBase (conserved miRNAs) and predicted in common between MIRcheck and MiRdup*. Ethidium bromide staining of the rRNAs is shown as gel loading control. L0 represents the control library for Al treatment (L8) in spring wheat Bounty which was not sequenced. The numbers between the parentheses correspond to the expression rank among the 199 predicted miRNAs. The lower value corresponds to the higher read abundance. For more information about the libraries and conditions see Additional file 1: Method S1 and Additional file 2: Table S1.

Selected miRNA candidates were validated by northern blotting, a useful criterion for authenticating miRNAs [51]. For this selection, miRNAs were ranked according to their expression level. Then, candidates were randomly chosen from either the predicted only by MiRdup* (three tested cases) or predicted by both MiRdup* and MIRcheck (six tested cases) as well as low, medium and high expression. Their characteristics and their secondary structures are presented in Figure 3a and Table 2. These structures reveal the less stringent rules in MiRdup* concerning the symmetric and/or asymmetric bulges in which the number of successive unpaired bases could range up to five nt in the duplex such as in apMiR_16808 (Figure 3a). Their expression was confirmed under all the investigated conditions (Figure 3b and c). Many probes detect more than one mature miRNA product with distinct lengths in different libraries, 19/21 nt for apMir_14769, 21/23 nt for apMir_20602, and apMir_22246 (Figure 3b and c). This indicates that the second detected miRNA product may be a variant of each of these miRNA candidates. At least two of these miRNAs exhibit complex expression patterns in response to cold, vernalization, salt, Al, and in development (Figure 3b). For instance, the larger miRNA product detected for apMir_14769 is preferentially expressed in the Al-treated library from spring wheat (L8). In addition, in some libraries the expression level of the apMir_20602 and apMir_22246 is much higher than what may be expected from the low read numbers obtained from deep sequencing (Figure 3c and Additional file 4: data SD1). This may be due to the presence of very closely related miRNA variants that can hybridize with the probes especially if the mismatches are at their start/end. Probes used would not be able to differentiate between these possibilities and thus would represent an average response of these related miRNAs. The miRNA size may affect an AGO1 functional state that mediates the recruitment of RDR6 [50,52]. However, for the apMir_20602 whose precursors overlap with transposable elements (Additional file 2: Table S5a), the high expression level and the presence of more than one size detected by northern may be associated with their repetitive nature with sequence variation in the genome.

**Table 2:** Characteristics of selected miRNAs using MiRdup* and MIRcheck validated by northern blot

**ID**	**MiRNAs**	**A**	**B**	**C**	**D**	**Associated conditions**	**Relevant targets (with uniref ID)**	**Training dataset**
Predicted MiRNAs in common with MiRdup* and MIRcheck								
apMir_20602	GUCAUCUAUAUUGGAACGGAG	1	1	1	5	Salt, floral transition and flowering	Glutathione peroxidase (Q9SME6), putative phosphatase phospho1 (M8CZ66)	BM
apMir_19980	AUAGCAUCAUCCAUCCUACCA	3	1	3	6	Al, floral transition and flowering	Putative membrane-associated protein (gi|22548307|gb|BU100508.1|BU100508)	BPM
apMir_14769	GUUGUCAUAUAUGUAUUGA	2	1	2	6	Cold, Al and salt	Putative RSH disease resistance-related protein (Q8H5X7), T-complex protein 1 subunit alpha (I3RZC6)	M
apMir_21052	UGAGAUGAGAUUACCCAAUAC	3	2	4	7	Cold, floral transition and flowering	NA	P
Predicted MiRNAs specific to MiRdup*								
apMir_16808	CAUCGAUCAUCCAUCACCC	2	5	6	7	Not differentially expressed	Dehydrin, (CD909074, TA50415_4565), Phosphorylase (Q84P16)	B
apMir_86202	AGGGUCGGCCAGCGGUGCGGCCCGU	4	2, 4	8	6	Cold, floral transition and Al	NA	BM
apMir_54471	UCAGUCAUAAUCCGGCAC	3	1	3	7	Salt, Al and floral transition	NA	MP
Conserved miRNAs predicted in common by MiRdup* and MIRcheck								
apMir_22246 **(tae-miR160)**	UGCCUGGCUCCCUGUAUGCCA	3	1	3	9	Cold, salt, Al, floral transition and flowering	Auxin response factor (M8BC98), Auxin responsive protein (R7WEP7)	BPM
apMir_20968 **(miR395-21)**	UGAAGUGUUUGGGGGAACUCU	2	1	2	8	Cold, salt, Al, flowering	Bifunctional 3′-phosphoadenosine 5′-phosphosulfate synthase (M7ZFX2), ATP sulfurylase (M9T1P9)	BMP

The selection was based on several characteristics of the miRNA secondary structure in the duplex including, the number of bulges in the duplex (A), number of the successive unpaired bases in each bulge in the duplex (B), total number of the unpaired bases within the duplex (C), nucleotide number in the loop (D). Training datasets (B: all miRBase species; P: all plants; M: monocot only). The reverse complement sequences of miRNAs used as probes for northern blot validation are presented in Additional file 2: Table S11.

### Expression of the identified miRNAs in response to different abiotic stresses and plant development in wheat

**Figure 4:**
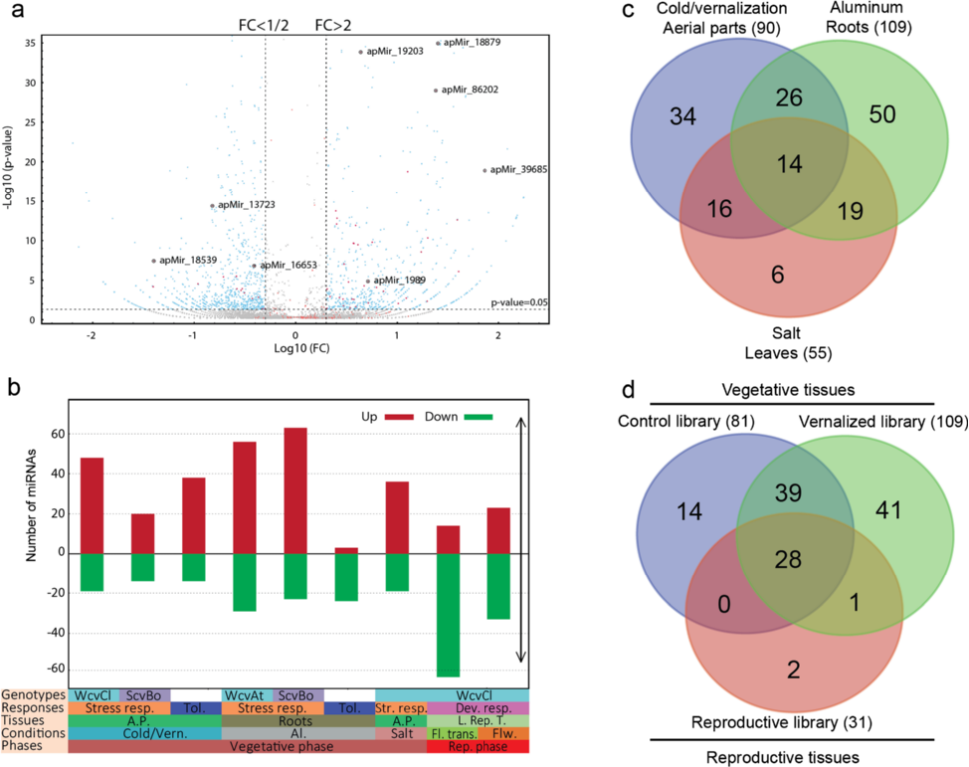
Differentially expressed miRNAs in response to cold, salt, aluminum and development. **a)** The differential expression of miRNAs in response to vernalization (presented on log10 adjusted p-value based on the FDR method of Benjamini and Hochberg [80], associated with the log10 of the fold change (FC)). The lines specify the thresholds used to identify the most relevant differentially expressed miRNAs. The blue and red dots correspond respectively to expressed small RNAs and predicted miRNAs; **b)** the frequencies of differentially expressed miRNAs in response to vernalization, cold, Al, salt and development stage (floral transition and flowering); and those differentially expressed between tolerant and sensitive genotypes; **c)** Venn diagram of miRNAs regulated under short/long exposure to cold (cold/vernalization, L2/L1 and L7/L6) in leaves, Al (L10/L9 and L8/L9) in roots and salt (L4/L1) in leaves; **d)** Venn diagram of miRNAs expressed in control plants during vegetative phase under normal conditions (control library L1), plants acclimated up to 56 days at 4°C (vernalized library L2) during vegetative phase and, plants acclimated up to 56 days at 4°C and then transferred to normal conditions under long day photoperiod to induce flowering during the reproductive phase (reproductive library L3). Up, up-regulated miRNAs; Dw, down-regulated miRNAs; Cold/vrn, cold and vernalization responsive miRNAs in spring (L7/L6) and winter wheat (L7/L6), respectively; salt responsive miRNAs in winter wheat (L4/L1); Al responsive miRNAs in spring (L8/L9) and winter (L10/L9) wheat; For tolerance, only differentially expressed miRNAs between cold (L2/L7) and Al (L10/L8) treated libraries are presented. All other abbreviations’ are described in the legend of Figure 2. See Additional file 1: Method S1 and Additional file 2: Table S1 about libraries and conditions and Additional file 2: Table S9 for more information about regulated miRNAs.

To identify miRNAs associated with short and long exposure to cold, salt and Al responses and tolerance, three different control and five treated libraries from sensitive and tolerant wheat genotypes were used. To identify miRNAs associated with floral transition and flowering in winter wheat, one library from plants at vegetative phase under normal growth conditions, one library under vernalization conditions (long exposure to cold acclimation) and one library from de-acclimated (one week under favourable conditions after cold acclimation) plants at the reproductive phase were used. Analysis of miRNA expression levels identified 91% (182/199) of miRNAs that are differentially expressed between the stress conditions compared to the control by more than twofold change with a FDR of 0.05 (see an example of volcano plot showing differential expression of miRNA candidates in response to long exposure to cold in the Figure 4a). Out of the 182 miRNAs, 165 miRNAs are responsive to different abiotic stresses (cold, Al and salt) and 99 miRNAs are associated with plant development, particularly floral transition and flowering (Figure 4b and Additional file 2: Table S9). Among abiotic stress responsive ones, 52 and 27 miRNAs are associated with cold and Al tolerance, respectively (Additional file 2: Table S9). We also find that regulated miRNAs may exhibit either common or specific expression patterns. Many of them show expression that is tissue, stress, genotype, or development stage-specific (Figure 4c and d). They may be specific to Al in roots, cold/vernalization and salt treatment in aerial parts, or common to two stresses or to all of the investigated abiotic stresses (Figure 4c). This indicates a crosstalk between the regulatory mechanisms of cold, Al and salt responses. This observation is confirmed by northern blot analysis showing a dynamic and complex expression pattern for several abiotic stress responsive miRNAs (Figure 3b and c). For instance, the candidates apMir_19980 and apMir_16808 are slightly up-regulated by cold, but also strongly down-regulated by salt and Al. The regulated miRNAs may be also specific to vegetative (L1- L2), reproductive phase (L3); or common to the two phases (Figures 3b and 4d). Moreover, out of the 199 miRNA candidates, less than 10% are ubiquitously expressed under the investigated conditions.

### Functional classification of abiotic stress and developmentally regulated miRNAs in wheat

**Figure 5:**
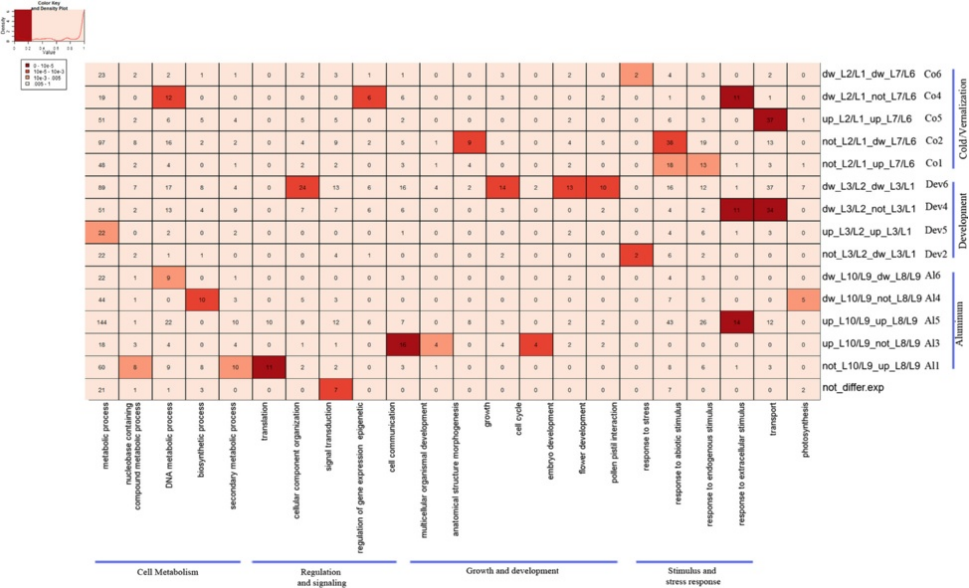
GO Slim enrichment for differentially expressed miRNAs in response to abiotic stress and development. Differentially expressed miRNAs with the same or different expression patterns between plants from tolerant and sensitive genotypes under normal and abiotic stress conditions; and between plants at vegetative and reproductive phases were classified into 24 miRNA groups. MiRNA targets are annotated to the best scoring GO Slim terms in Biological process category. The lines are grouped according to their association to cold and vernalization (L2/L1 and L7/L6), Aluminum (L10/L9 and L8/L9) and development (L3/L1 and L3/L2). See Additional file 2: Table S10 for more information about miRNA groups. The value in each case indicates the number of associations miRNA-target- GO for the corresponding GO Slim. The enrichment is presented in four different colors (“brown square symbol” high enrichment (P-value < 10^−5^), “orange square symbol” medium enrichment (P-value < 10^−3^), “light orange square symbol” low enrichment (P-value < 0.05) and “white square symbol” no enrichment (P-value ≥ 0.05).

The potential functions of regulated miRNAs (differentially expressed between two conditions) were classified into 24 miRNA groups for cold (Co1-Co8), Al (Al1-Al8) and development (Dev1-Dev8) according to their expression in two wheat genotypes that differ in their degree of tolerance as well as during different development stages (Additional file 2: Table S10). For each stress, we found that groups 5 and 6 (Co5-Co6 for cold/vernalization; Al5-Al6 for Al) having similar expression profiles in tolerant and sensitive genotypes are associated with cold/Al responses while other groups (Co1-Co4, Co7-Co8 and Al1-Al4, Al7-Al8) showing different expression patterns between the two genotypes are associated with tolerance (Additional file 2: Table S10). For development miRNA groups, 6 groups are associated with floral transition (Dev3-Dev4) and flowering (Dev1-Dev2 and Dev7-Dev8). The 24 miRNA groups were subjected to GO enrichment analysis based on the 3 categories: cell component, molecular function and biological process (Additional 3: Figures S4b-6Sb). Several highly enriched GO Slim terms associated with the studied conditions (Figure 5 and Additional file 3: Figures S4b-6b).

The miRNA group associated with cold responses (Co5) is specifically enriched for *membrane* (triose phosphate translocator) in the category cell component (Additional file 3: Figure S4b) and *signal transduction *(*Auxin-responsive proteins*) in the category biological process (Figure 5 and Additional file 3: Figure S6b). Consistently, enrichment is also found for nucleus (Additional file 3: Figure S4b), *protein binding activity* (Additional file 3: Figure S5b), *nucleobase containing component metabolic process and response to endogenous stimulus* (Figure 5 and Additional file 3: Figure S6b) which are all overrepresented by auxin responsive proteins. These results indicate that cold regulated miRNAs may function in carbon partitioning during photosynthesis and in auxin-activated signaling pathways. For miRNA groups associated with Al responses (Al5-Al6), an enrichment is found for *hydrolase activity* (protein phosphatase 2C, lipase)*, catalytic activity* (glutathione peroxidase, phenylalanine ammonia-lyase) (Additional file 3: Figure S5b), *response to endogenous stimulus* (Auxin Response Factors), *DNA metabolic process* (histone 4) (Figure 5 and Additional file 3: Figure S6b). These results indicate that Al-regulated miRNAs may function in regulation of gene expression and signaling as well as plant defense under oxidative stress. More interestingly, many targets found for miRNA groups associated with cold (Co1, Co2, Co4) and Al (Al1, Al2 and Al3) tolerance are known for their function in stress adaptation. For miRNA groups associated with cold tolerance, the groups Co1 and Co2, showed enrichment for the GO Slim term *response to stress* (phosphoglycerate mutase, Defensin-like protein 1, Universal stress protein A-like protein). In addition, Co1 is enriched for *response to abiotic stimulus* (thaumatin-like protein, glutathione S-transferase) (Figure 5 and Additional file 3: Figure S6b) and the group Co2 is enriched for *cell wall* (Defensin-like protein, phospho-3-sulfolactate synthase-like), *nucleus* (CBFIVb-B20), *hydrolase activity* (Ubiquitin-like-specific protease, Serine carboxypeptidase)* and catalytic activity* (Gibberellin 20 oxidase, Glyceraldehyde-3-phosphate dehydrogenase) (Additional file 3: Figure S5b)*.* This indicates that the identified cold regulated miRNAs may function in proteolysis, gibberellins biosynthesis and glucose metabolism. The group Co3 is enriched for *transporter activity* (Sodium/hydrogen exchanger) (Additional file 3: Figure S5b) while the group Co4 is enriched for *pollen-pistil interaction* (Serine/threonine-protein kinase) (Figure 5 and Additional file 3: Figure S6b) indicating that they may also have a function in ion transport and signaling.

Moreover, for groups associated with Al tolerance, the most enriched GO Slim terms are *mitochondrion, transporter activity* (Cytochrome b-c1 complex) and *carbohydrates metabolic process* (glyceraldehyde-3-phosphate dehydrogenase) indicating that miRNAs from these groups may mediate glycolysis and respiration process under Al stress conditions. Enrichment is also found for the terms *catalytic activity, hydrolase activity, sequence specific transcription factor activity* (Additional file 3: Figure S5b), *secondary metabolic process, embryo development*, *anatomical structural morphogenesis* and *multi-organismal development* (Figure 5 and Additional file 3: Figure S6b). They are overrepresented by many important metabolism enzymes involved in phenylpropanoid metabolism and ubiquitination process (phenylalanine ammonia-lyase, DEAD-box ATP-dependent RNA helicase, Ubiquitin carboxyl-terminal hydrolase*)* as well as transcription factors (Homeobox-leucine zipper proteins).

Interestingly, the targets in miRNA groups associated with development are involved in cell growth and flowering. The miRNA groups associated with flowering (Dev1, Dev2) show enrichment for the GO Slim terms *protein binding activity, sequence specific DNA transcription binding activity* (MIKC-type MADS-box transcription factors), and/or *kinase activity* (Serine/threonine-protein kinase) (Additional file 3: Figure S5b). They are also enriched for *nucleobase containing compound metabolism* and *pollen pistil interaction* that are specifically represented by two well characterized regulators genes (MIKC-type MADS-box and Serine/threonine-protein kinase) (Figure 5). One miRNA group associated with floral transition (Dev 4) is enriched for the GO Slim terms *transport* and *response to endogenous stimulus,* specifically represented by Auxin-responsive proteins. Furthermore, the potential function of ubiquitously expressed miRNA libraries was also investigated. They show significant enrichment for the GO Slim terms *thylakoid* (Additional file 3: Figure S4b), *kinase activity*, *nucleotide binding activity* (Additional file 3: Figure S5b), and *cell protein modifications* (Figure 5 and Additional file 3: Figure S6b). This result indicates that constitutively expressed miRNAs may modulate the basic cellular functions reflecting their vital regulatory role in other growth conditions yet to be identified in wheat.

## Discussion and conclusions

### The wheat miRNA pipeline

In this study, we developed a pipeline that identifies conserved as well as clade and species-specific or young miRNAs. This pipeline can be easily adapted for other plant species. To predict miRNAs from NGS and analyze their function, the steps described in Figure 1 are required. While several steps are standard in NGS analyses [4,49,53], we improved the miRNA prediction steps by integrating folded pre-miRNA candidates, expression profiling and functional analyses of differentially expressed candidates. To address the step of miRNA prediction, we decided to exploit two methods with different algorithmic schemes MiPred [43] and HHMMiR [44] to have a broad range of hairpin candidates. These methods were trained on pre-miRNAs from plants and wheat sequences available in miRBase and resulted in the identification of a large number of pre-miRNA candidates using the predictors MiPred [43] and HHMMiR [44]. To address issues of latter methods for the lack of consideration of mature miRNA and their surrounding biological features, we developed a classifier that ranked the best 35 biological features of plant miRNAs that was integrated into MiRdup* (Additional file 2: Table S3). For robustness, the classifier’s models were trained separately on three datasets (all miRBase species, all plants and only monocots). This increases species-specificity and allows the discovery of features that distinguish wheat miRNAs from those of other species. The developed classifier (MiRdup*) was able to reduce the level of false prediction obtained by MiPred [43] and HHMMiR [44] by more than 81% (Figure 1, Additional file 3: Figures S1 and S2) and allowed the assessment of the position of a miRNA in a given pre-miRNA sequence. In addition, the combinatorial analysis between MiRdup* and MIRcheck [45] which identifies 20-nt regions of a given plant pre-miRNA using a predetermined set of rules and constraints, show that MIRcheck is too stringent and easily removed experimentally validated miRNAs (Figure 3b and Additional file 3: Figures S2).

The availability of wheat EST databases and our approach enabled us to identify with confidence 199 miRNA candidates. These candidates may include miRNA gene homeologs from the three genomes of hexaploid wheat, or ESTs with SNP differences in different wheat varieties. It is also reasonable to assume that these families represent only a fraction of the total miRNAs that may exists in hexaploid wheat since many small RNAs still remain unmapped to wheat sequences or conserved miRNAs from miRBase. The availability of the complete assembled and well-annotated hexaploid wheat genome will help to complete the discovery of the remaining miRNAs.

It is important to emphasize that among the predicted miRNAs, in spite of being derived from ESTs, less than 5% of the mature miRNAs are associated with known protein coding regions and less than 7% are related to transposable elements (Additional file 2: Table S5a and S5b). According to Dinger *et al*., [54], many transcripts are categorized as bifunctional RNAs. They can be translated into protein but also function independently as RNA. The presence of such bifunctional RNAs challenges the assumption that the RNA world can be neatly parsed between mutually exclusive protein-coding and non-coding categories.

### MiRNA candidates associated with abiotic stress responses

This study represents one of the largest *de novo* miRNAome analyses in response to different abiotic stresses and development in hexaploid wheat. Although many cold responsive miRNAs have been identified in spring wheat using NGS [6], our study identified a large number of novel candidates regulated by cold, vernalization, Al and salt with dynamic and complex expression patterns (Figures 3b, 4b and Additional file 2: Table S9). Several identified miRNAs are either associated with a specific stress or common to at least two stresses (Figures 3b and 4c). Many of their targets are known to be stress-related genes (Figure 5 and Additional file 3: Figures S4b-6b) commonly regulated under abiotic stresses.

Our results show that miRNAs may mediate plant responses to Al treatment by regulating expression of stress related genes particularly those involved in auxin signaling and fatty acid metabolism. This is consistent with the fact that Al affects the relative abundance of membrane lipids and the degree of fatty acid unsaturation [55,56] and Auxin Response Factors (*ARFs*) that are known to inhibit root development in response to Al toxicity [57]. In addition, the experimentally validated apMir_22246 (which corresponds to *tae-miR160*) is regulated by Al exposure (Figure 3c) and targets specifically ARFs. Many ARF members are known to be regulated by *miR167 and miR160* and to play regulatory roles in adventitious rooting [58], supporting the possible role of apMir_22246 in root development under Al treatment.

### MiRNA candidates associated with cold responses and freezing tolerance

Our data indicate that cold regulates the expression of several miRNAs in spring as well as in winter wheat (Figures 3b, 4b and c). Four miRNA groups associated with cold tolerance (Additional file 2: Table S10) target a set of cold regulated genes known to be involved in freezing tolerance including the transcription factors *CBFs*, dehydrins, DEAD-box RNA helicases, thaumatin-like protein [59-62]. Interestingly, many candidate miRNA target genes related to the ICE1–CBF major pathway that regulates freezing tolerance in cold hardy plants. This includes the targets DEAD-box ATP-dependent RNA helicase 12, CBF and dehydrin (Additional file 2: Table S7). Results from our previous studies demonstrated that genes related to the ICE1–CBF pathway play a critical role in freezing tolerance in hexaploid wheat [63]. Here we show that the miRNA candidate apMir_16808 is regulated in response to cold (Figure 3b), and target the cold responsive genes dehydrins (Table 2) [59,62]. The candidate apMir_19532 from miRNA group associated with cold tolerance target CBFIVb-B20 gene (Additional file 2: Table S7). These results suggest that these miRNAs may contribute to freezing tolerance by regulating cold-regulated genes belonging to the CBF regulon in winter wheat.

### Predicted miRNA target genes common in regulating several stresses

Plants evolved common regulatory mechanisms to adapt to environmental stresses such as oxidative stress commonly induced by both cold and Al. Our results show that many of the identified abiotic stress responsive miRNAs exhibited a common stress expression pattern (Figures 3b and c and 4c). For instance, the expression of the new member of miR395 family, miR395-21 corresponding to apMir_20968, is commonly regulated in response to cold and Al stress (Figure 3c) indicating that miR395 is not specific to sulfate starvation as previously reported in *Arabidopsis* and rice [49,64]. Zhao *et al*., [65] also reported that miR395 is involved in phosphate homeostasis in wheat. This indicates that miR395 mediates not only plant response to sulfate deficiency but also may mediate responses to other nutrients that are imbalanced under abiotic stress conditions. Taken together, our results indicate that miR395 would play a common role in plant nutrient homeostasis under abiotic stress conditions. In agreement with previous suggestions, our results indicate that miRNAs coordinate crosstalk among different nutrient deficiencies. This is the first indication that crosstalk between cold, Al stress and plant nutrients could be regulated by miRNAs. Moreover, we show that the miRNA candidate apMir_20602 is also commonly regulated under cold, salt and Al (Figure 4b) and targets glutathione peroxidase (Table 2). Recent findings showed that human miRNAs regulate glutathione peroxidase expression to maintain redox homeostasis [66]. This supports the possible role of apMir_20602 in mediating crosstalk between abiotic stress responses by regulating glutathione metabolism.

### Wheat vernalization responsive miRNAs associated with floral transition and flowering

In this study, we investigate the role of miRNAs during the transition from the vegetative to the reproductive phase, and during flowering in winter wheat that requires vernalization to flower. We found that among developmentally responsive miRNAs, many candidates target cold responsive genes known for their function in flowering transition and flower development (Additional file 2: Table S7). For instance, the candidate apMir_19892 corresponding to hvu-miR444b (Additional file 4: data SD1) could target many MIKC-type MADS-box transcription factors*, t*he homologs of *TaAGL17* and *OsMADS57.* In wheat*,* MIKC-type MADS-box transcription factors control flower development and morphogenesis [67]. In barley, this target contains both the target site for miR444b and the precursor sequence for miR444a [68]). In rice, *OsMADS57* is involved in axillary bud development and regulation of tillering through down-regulation of miR444a [69]. Since the miRNA variants from miR444 family are functional, and *MADS*-box genes are collectively regulated by the miR444 family [70], we suggest that apMir_19892 may mediate flowering through the regulation of MIKC-type MADS-box transcription factor gene expression. ApMir_19532 target genes encoding Ubiquitin-like-specific protease *ESD4* known to regulate plant responses to cold and the time of flower initiation [71,72]. In addition, apMir_20860 corresponding to miR159 (Additional file 4: data SD1) are involved in promotion of floral transition in many species*.* In ornamental plants, miR159-regulated *GAMYB* expression is an effective pathway of flowering time control [73]. This suggests that apMir_19532 and apMir_20860 may mediate flowering time in wheat through the regulation of Ubiquitin-like-specific protease ESD4 and *GAMYB* gene expression.

## Methods

### Plant material and small RNAs isolation

In this study, three genotypes of hexaploid wheat (*Triticum aestivum L*. 2n = 6x = 42, AABBDD), one spring genotype (cv Bounty, cold and Al sensitive) and two winter genotypes (cv Clair, cold tolerant and Atlas66, Al tolerant genotype) were used to construct ten different small RNA libraries from plants in vegetative and/or reproductive stages and/or exposed to different stress treatments or under normal conditions (Additional file 1: Method S1 and Additional file 2: Table S1). To identify miRNAs that are associated with different development stages, tissues from both vegetative and reproductive phases were used. Vegetative phase samples include leaves and crown from the aerial part of plants. Reproductive phase samples include leaves at flag leaf stage, developing spikes with sizes ranging from 2 to 110 mm, and spikes partially and completely-opened with and without pollen. We used also root tips to identify miRNAs associated with Al and salt stress, and aerial parts including leaves and crown to identify miRNAs associated with cold and salt. In addition, we used different genotypes of winter (tolerant) and spring (sensitive) wheat to identify miRNAs associated with Al and freezing tolerance. Small RNA extraction was initiated from 200 mg of a mixture of leaves, stem or root tip tissues from 10 to 100 seedlings for each time point. Control and treated plants were sampled at the same time of the day for each time point (except for the first day where a few samples were taken at short time points) as described in (Additional file 1: Method S1 and Additional file 2: Table S1). Small RNAs (below 200 nt) were isolated from each sample using the mirVana miRNA Isolation Kit (Ambion Inc. US). MiRNAs (small RNAs below 40 nt) from each time point were isolated using the flashPAGE fractionation kit (Ambion) (Additional file 1: Method S1 and Additional file 2: Table S1), and then purified using the flashPAGE Reaction Clean-up kit (Ambion) according to the manufacturer’s protocols. Their integrity was assessed using a DNA 1000 LabChip on an Agilent 2100 Bioanalyzer (Santa Clara, CA, USA).

### MiRNA libraries construction and sequencing

Twenty five nanograms of purified miRNAs from each time point of a given condition (Additional file 1: Method S1 and Additional file 2: Table S1) were pooled and used as a template to produce the corresponding miRNA library. MiRNAs were tagged with a barcode system containing ten unique and specific amplification primers (1 barcode/library) and ten cDNA libraries were produced using the SREK kit (small RNA expression Kit, Ambion) according to the manufacturer's protocol. The libraries were sequenced on the SOLiD Analyzer according to the standard protocol (V2.1 Applied Biosystems).

### Experimental validation of predicted miRNAs

For each library, identical amounts of plant tissues from each time point were ground and mixed with TRIzol Reagent (Life Technologies). The same extract volume from each time point of each library was pooled to isolate small RNAs using the mirVana miRNA Isolation Kit. Five micrograms of small RNAs from each library were analyzed by northern blot [74]. The experiment was repeated at least twice for each selected probe. The oligonucleotide probes are presented in Additional file 2: Table S11.

### Identification and extraction of potential pre-miRNA candidates from sequenced small RNAs

From the 89 million reads obtained from the ten libraries, we first removed reads that have low quality scores as recommended for SOLID sequencing [39,75] (Additional file 2: Table S2). After adapter removal using the program cutadapt v0.9 [76], small RNAs between 18 and 30 nt were mapped to ESTs with the MAQ v07.1 program [77] allowing a maximum of two mismatches (Additional file 1: Method S3). Then, sequences with low complexity or containing repeats were filtered out using RepeatMasker v3.2.9 [78] with RepBase15.09 and Repeatmasker-Libraries-20130422 [79], and a slow search method against *Triticum aestivum* and *Oryza sativa*. For each mapped EST, we considered two sequences that could include a potential pre-miRNA candidate as follows: 20 nt before the start and 160 nt after the end of the mapping were extracted from both EST strands.

The secondary structures of the extracted sequences (pri-miRNA) were folded with RNAfold from ViennaRNA v1.8.4 package [80] to identify those having a hairpin-like shape, one of the fundamental characteristics of pre-miRNAs. Then, these sequences were submitted to two pre-miRNA predictors using different algorithmic schemes, HHMMiR [44] and MiPred [43], which were trained with pre-miRNAs of all cloned or sequenced miRNAs from miRBase [30]. Finally, to identify conserved miRNAs, we performed a *blastn* of small RNAs with more than 100 reads in at least one library against miRBase (V21) [81] with a *word_size* 7, *maximum e-value* 0.1, *percentage identity* 80, *gap open penalty* 5, *gap extension penalty* 2, *match score* 1, *mismatch score −*2, *filter low complexity*. We restricted the blast results as follows: query coverage of 90%, subject coverage of 90%, with no gap allowed.

### Filtering false positive pre-miRNAs

We adapted our previously published machine learning classifier [41] to the best plant features associated with the position of miRNA-miRNA* duplex in the pre-miRNA (Additional file 1: Method S4), and we named this version MiRdup*. The latter differs from the original one on the 35 retained features (Additional file 2: Table S3) that are relevant for plants and has been trained on all experimental (cloned or sequenced) miRNAs from miRBase subdivided in three datasets (all species, all plants and only monocots). This classifier computes a score of prediction scaled between 0 and 100 (more evidence). The potential pre-miRNAs from MiPred or HHMMiR that obtained a MiRdup* classification score higher than 90 and with miRNA read abundance above 100 in at least one library were selected as candidates. In addition, to help identifying the potential functional miRNAs among several candidates, we applied the relaxed expression rules derived from the update of the specific criteria for plant miRNA annotation reported by Meyers *et al*., [42]. In parallel, we applied MIRcheck [45], a well-known tool for plant miRNA identification, on the overall predicted miRNAs to compare the differences with MiRdup*. A combinatorial analysis between the two tools is provided (Additional file 3: Figures S1 and S2). Then, the pre-miRNAs were blasted against TREP database to identify miRNAs that originate from transposable elements (Additional file 2: Table S5a). The e-value threshold used is 5.0E-05 and Hit Coverage (HC) ≥ 85 and percentage of identity ≥80. We performed also a blastx of ESTs producing the identified pre-miRNAs against proteins coding sequences from protein plant database with default parameters (Additional file 2: Table S5b). The threshold to include a blast hit of an EST into a given protein core is an e-value lower than 1.00E-20 and query coverage or hit coverage higher than 85% and percentage of identity higher than 75%.

### Statistical analyses of the abundance of potential miRNAs

To quantify and compare sequence abundance across different libraries, raw read counts were normalized using rpm (reads per million). Sequences with read counts lower than 100 in all libraries are removed. Significance level of the difference of small RNA between two libraries was analyzed using a corrected Z–Score method as described in Kal *et al*., [82]. An adjustment for multiple comparisons based on the false discovery rate (FDR) [83] was performed (FDR < 5%). The small RNAs with fold change lower than 0.5 or higher than 2.0 were retained.

### MiRNA target analyses and GO enrichments

MiRNA target genes were identified using the FASTA engine of Tapir v1.0 program, with a stringent maximum score of three and minimum free energy ≥ 0.7 [84] excluding ESTs annotated as unknown proteins. For the obtained and annotated target genes, we retrieved their classification in Gene Ontology (GO) through the GO Slim viewer on AgBase webserver [85]. The GO Slim enrichments were performed using the standard hypergeometric test. The wheat genome GO Slim background was constructed taking into account the overall GO Slims covered by the 127,039 UniRefs id retrieved from all the collected ESTs Database. The GO Slim terms with P-value < 0.05 were considered as enriched. The same procedure was applied for the targets of the differentially expressed miRNAs. Unlike the overall analysis, the GO Slim background considered in each condition was computed from only the GO Slim of the identified target genes present in the two compared libraries from sensitive and tolerant genotypes. For functional analysis, we investigate the potential function of all identified miRNAs in the 10 investigated conditions (10 libraries) for miRNAs having at least 100 reads in at least one sequenced library. (Additional file 4: data SD3).

### Availability of supporting data

All the predicted and conserved miRNAs from this study, the published miRNAs from the literature, the small RNA expression profiles are provided at the following database http://wheat.bioinfo.uqam.ca.

## Additional files

Additional file 1:
**Supplementary Methods from S1 to S4; Provides exhaustive description of different section of the methods.**
Additional file 2:
**Supplementary Tables from S1 to S11; Provides supplementary Tables of the manuscript and their associated legends.** Long Tables are given xls files embedded in the zip but their legends remain in the main supplementary table files named < Tables S1to5 and S8to11.docx >. Additional file 3:
**Supplementary Figures from S1 to S7; Provides list of supplementary figures.**
Additional file 4:
**Supporting Data from S1 to S3; Provides supporting Data of the manuscript and their associated legends.** Their legends are in the main supplementary files named. < Supporting Data_legendeSD1toSD3.docx>. The three datasets present an elegant representation of the miRNA within their coexpressed small RNAs, their percentage of coverage and the list of miRNA with more than 100 reads. Additional file 5:
**Appendix-GO enrichment all targets.pdf.** Gene ontology enrichment for the predicted target genes; Description of the data: Summary of all the enriched Go terms.
