# Supplementary material for: An integrative approach to identify hexaploid wheat miRNAome associated with development and tolerance to abiotic stress
Source: BMC Genomics. 2015 Apr 24;16(1):339. doi: 10.1186/s12864-015-1490-8 (PMC4443513; doi:10.1186/s12864-015-1490-8)
Supplement: Additional file 1: — Supplementary Methods from S1 to S4; Provides exhaustive description of different section of the methods. [file 12864_2015_1490_MOESM1_ESM.pdf]

Additional file 1. Supplementary Methods from S1 to S4

Method S1: Plant treatment

To identify miRNAs associated with stress responses and tolerance, a control library from plants grown under normal conditions was constructed except for the Al treated library from spring wheat Bounty (sensitive genotype). We used the control library from winter wheat Atlas66 (the tolerant genotype) as a control since in our previous study; the expression level of most genes was similar in control plants from Atlas66 and Bounty. Only 1.26% of genes were differentially expressed in the microarray analysis between Atlas66 and Bounty control demonstrating that most genes are expressed at a similar level (Houde and Diallo, 2008). For cold and salt treatments, seeds were grown in 3 L pots containing mixed soil (compost, black earth, vermiculite, 1: 1: 1) at 20°C under a 16 h photoperiod with a light intensity of 250 µmol m^{-2} s^{-1} and 70% relative humidity. To control the soil water content, one week before starting treatment, all plants were watered with the same amount of water, so they were exposed to the same soil moisture content. Control untreated plants were kept under the same conditions (normal temperature and watering) for the duration of the experiment, while treated plants were subjected to different abiotic stresses for various periods of time. For salt treatment, four week-old plants were watered daily with the same volume of a 200 mM NaCl solution and sampled at days 1, 3, 5, 7, 12 and 15. For cold acclimation, two week old plants were transferred to a 4°C growth chamber in the same conditions of light and watering, and sampled after 1, 2, 7, 14, 21, 28, 35, 49 and 56 days. For Al treatment, seeds were grown in moist vermiculite during 5 d at 20°C under continuous light: 25 µmol m^{-2} s^{-1}. Seedlings were exposed to Al stress as described in Hamel *et al*., (1998) using 5 µM Al for Bounty and 50 µM Al for Atlas66.

Method S2: Reference genome

We collected the Expressed Sequenced Tags (ESTs) from different databases: GrainGenes (Carollo *et al.,* 2005), TIGR (Childs *et al.*, 2007), WheatDB (wheatdb.ucdavis.edu:8080/wheatdb/), TAGI (compbio.dfci.harvard.edu/tgi/), Komugi (www.shigen.nig.ac.jp/wheat/komugi/) and from our database (Houde *et al.,* 2006). These data were clustered according to Uniprot UniRef100 clusters to reduce the noise given by partially and nearly identical ESTs. To cluster the ESTs, each one has been aligned with the program *blastx* (Gish and States 1993) with default parameters. Each EST is annotated with a Uniref100 protein if it has at least 60% identity, 60% query coverage and/or hit coverage and E-value inferior to 5E-5.

Method S3: Removing adaptor and read mapping

All sequences are from SOLID sequencing and had a length of 35 with T as first character. Since expected mature miRNA length is around 21nt, reads should contain a part of the adaptor P2. Adapters were removed in color space (represented by numbers between 0 and 3) using the program cutadapt v0.9 (Schulte *et al.*, 2010) with the following parameters: -c -e 0.12 -a 330201030313112312 –maq. Those parameters accept at most 12% of mismatches over

the adaptor, therefore adjusting the number of mismatch depending on sequence length. The output format was adapted to the program MAQ (Ondov *et al.,* 2008) for mapping the adaptor free reads to the ESTs. Identical or different reads with the same mapping results (same region in the EST) have then been grouped as a small RNA. We computed the abundance of a small RNA as the sum of all reads that exhibit the given small RNA after mapping.

Method S4: MiRdup* **model creation**

S4.1 Collected data

All miRNAs and pre-miRNAs were downloaded from miRBase release 18, which contains 21643 miRNAs or miRNAs* for 18226 pre-miRNAs. The negative dataset was generated within the same data, except the start position of the miRNA was chosen randomly on the pre-miRNA. The different lengths were preserved. The negative miRNA cannot be equal to the original miRNA and cannot exist in the positive training dataset. The complete training dataset has 46412 instances, so 23206 instances for each positive and negative dataset. This equal representation avoids any influence by one or the other datasets on the model.

In order to test our classifiers, we validated them on miRBase and a few other datasets. The first dataset, Plant miRNAs Database (Zhang *et al*., 2010), which contains 10081 instances of combination miRNA and pre-miRNA. Two other datasets come from papers which can analyze real data using different ways to predict miRNAs: MIRcheck (Jones-Rhoades & Bartel 2004) with 187 instances and miRDeep (Friedlander *et al*., 2008) with 228 instances.

S4.2 Model creation

To perform the feature selection and create models, we used Weka and its libraries (Hall *et al*., 2009). For all trained models, we applied a 10 fold cross validation.

In MiRdup*, we chose the combination of Adaboost M1 method (Freund & Schapire 1995) and forest of random trees (Breiman 2001). Adaboost is a machine learning algorithm specialized in problem optimisation and the search for the best global optimum (Osman 1996). It is used in combination with many other machine learning algorithms in order to improve their performances. We trained it with 10 iterations, reweighting, and a weight threshold of 100.

Concerning other classifiers, the SVM classifier, working with libSVM library (Chang and Lin 2001), was trained with radial kernel and the C4.5 tree (Quinlan 1993) was trained with Adaboost.

Sensitivity and specificity, which measures the proportion of positives and negative instances which are correctly classified as such respectively, were calculated with the well-known formulas:

$sensitivity = \frac{truepositives}{truepositives+falsenegatives}$ and $specificity = \frac{truenegatives}{truenegatives+falsepositives}$

We set 35 features that can characterize the duplex miRNA and its complement sequence on the hairpin (Additional file 2: Table S3). To calculate them, our model requires the mature sequence of the miRNA, the pre-miRNA hairpin loop sequence and its secondary structure. Few are based on the position and length of particular structural elements in the miRNA, like bulges and bases pairs, or the situation of the miRNA itself in the hairpin, like the distance relative to the loop. We also included features that characterize the nucleotide sequence, where we check for missing nucleotides in the miRNA, percentage of presence of certain nucleotides and GC content. Furthermore, we calculate the minimum folding energy of the duplex.

Those features reveal some configuration errors found in pre-miRNA prediction results. In Figure SM1b, we show examples of misplaced miRNAs (red) on a predicted pre-miRNA (black), where the miRNA sequence might extend in the loop or in a big bulge. We assume that could affect the biological process and thus, conclude that the miRNA cannot be handled by the given pre-miRNA. Those should be invalidated. On the opposite, miRNAs (yellow) which have a good position on the hairpin are validated, depending of the training set characteristics.

S4.3 Features ranking

Not all features have the same impact on classifiers. Some are too similar between the positive and negative datasets and can influence badly the classifier, especially in over fitting and computational time (Zhou *et al.*, 2006). Then, in order to get the most important features, we ranked them, as shown in Additional file 2: Table S3 for all the miRBase dataset. We observe that the most influent feature concerns the distance from the terminal loop and the weakest is the presence of U. In addition, presence and percentage in the miRNA of a certain nucleotide tend to be less influential features. The miRNA length is a positive control and has no difference between positive and negative datasets, because generation of the negative dataset is done by moving the miRNA while preserving its length. As shown by Leclercq *et al.*, (2013), depending on the kingdom of species chosen for the study, the rank of important features will change. Hence, to ensure quality, we trained the model on all miRBase, all plants and monocot datasets.

S4.4 Training models on classifiers

Various classifiers were tested in order to find the best one which could discriminate all experimentally validated miRNAs from miRBase dataset depending on the class (Table SM1): SVM trained with radial basis kernel, boosting with Adaboost on C4.5 tree and Adaboost on forest of random trees. Two sets of features were experienced on the classifiers: best features and all features. Best overall scores are given by Adaboost on forest of random trees on all 35 features, with 80.23% correctly classified instances and 86.5% sensitivity (SE). Only specificity (SP) with 74.1% is less than with others classifiers. SVM showed 93.5% SP, but this classifier could only classify instances correctly as 71.81% and has a poor SE of 50.2%. Concerning boosting on C4.5 tree, results are close to forest of random trees, but are always slightly below. However, we noticed that SVM can get better scores when using only the best features set, and this improves correctly classified instances to 75.77% and SE at 71.1%. However, this is still less than forest on random trees with all features. It's important to note that only SVM improves its results with only best

features, which is the opposite with other tested classifiers. Also, a disadvantage of the SVM is that it takes a very long time to train on all miRBase data, several hours compared to few minutes for other classifiers. Hence, the best compromise would be Adaboost on Random Forest.

Table SM1: Results of various classifiers on the all miRBase training dataset with 10 fold cross validation. Best scores for a classifier in each category are highlighted in bold. **a.** Classifiers trained on all 35 features. **b.** Classifiers trained on the 14 best features.

a)

Classifier	Correctly classified instances	Correctly classified instances (%)	True positive rate	False positive rate	True negative rate	False negative rate	Sensi tivity	Specifi city
**SVM radial basis kernel**	33332	71.81	0.502	**0.065**	**0.935**	0.498	0.502	**0.935**
**Adaboost on C4.5 tree**	36780	79.24	0.814	0.229	0.771	0.186	0.814	0.771
**Adaboost on Random Forest**	**37262**	**80.28**	**0.865**	0.259	0.741	**0.135**	**0.865**	0.741

b)

Classifier	Correctly Classified Instances	Correctly classified instances (%)	True positive rate	False positive rate	True negative rate	False negative rate	Sensi tivity	Speci ficity
**SVM radial basis kernel**	35170	75.77	0.711	**0.196**	**0.804**	0.289	0.711	**0.804**
**Adaboost on C4.5 tree**	36157	77.90	0.802	0.244	0.756	0.198	0.802	0.756
**Adaboost on Random Forest**	**36646**	**78.958**	**0.853**	0.274	0.726	**0.147**	**0.853**	0.726

S4.5 Evaluation of the designed models

After training the classifiers on all features, few datasets were submitted to construct the models. These include miRBase and plant miRNAs (PMRD) databases, and published results from MIRcheck and miRDeep. Results are

shown in Table SM2, where we have the total instances of every dataset, the portion of validated instances and the mean absolute error. An instance is a miRNA and its corresponding precursor. The mean absolute error informs about the average error per predicted instance made by the model. We note in the Table SM2 that the mean absolute error follows the percentage of validated instances. We also observe that Adaboost on forest of random trees model have the best scores of validated instances and the lowest mean error rate in every dataset, except MIRcheck, where Adaboost on C4.5 model validated 4 more instances. The most interesting is miRBase dataset, which is our reference, where Adaboost on forest of random trees validated 99.75% of the 23206 miRNAs with their precursors, with a very high confidence score (lowest mean absolute error). PMRD, which contains a lot of computational predictions, has only 78.18% of validated instances and makes models less confident with their predictions than the miRBase dataset. On the other hand, we can assume that MIRcheck already does very good predictions, with 94.65% of validated instances with Adaboost on C4.5 model, which is not the case of miRDeep, the best score of validated instances is 84.51%, predicted with Adaboost on forest of random trees model. Finally, SVM has lower scores than other models. Since we used the model trained on all features, which does not advantage SVM, results are improved if the model is trained on best features, but not enough to beat other classifiers on the miRBase dataset: 97.97% of correctly classified instances (22736 instances) and a mean absolute error of 0.0203.

Table SM2: Evaluation of chosen models trained on all features. The evaluation is done on predicted hairpins composed of predicted pre-miRNAs with their sequenced miRNAs. Data comes from miRBase, PMRD, MIRcheck and miRDeep.

**Dataset**<br>**Total instances**	**miRBase**<br>**23206**		**PMRD**<br>**10081**		**MIRcheck**<br>**187**		**miRDeep**<br>**226**	
**Model**	Validated instances	Mean absolute error	Validated instances	Mean absolute error	Validated instances	Mean absolute error	Validated instances	Mean absolute error
**SVM radial basis**	96.51%	0.03	36.91%	0.63	59.35%	0.40	52.21%	0.47
**Adaboost on C4.5 tree.**	99.49%	0.005	72.32%	0.28	**94.65%**	0.06	80.53%	0.20
**Adaboost on RandomForest**	**99.75%**	0.002	**78.18%**	0.21	92.51%	0.07	**84.51%**	0.16

S4.6 Conclusion of the MiRdup* model

We trained several classifiers, and boosting on forest of random trees showed best overall results. It has the best classification score of 80.29% and ability to identify negatives results (specificity) 86.5%. Its sensitivity is not the top, but still gives a high score of 74.1%. The evaluation of miRBase on this model has shown a high score of 99.75% validated instances of mature miRNAs and corresponding pre-miRNA(s).

It's not the first time where SVM, Adaboost, trees and random forest are used in this field to classify real or false miRNAs and pre-miRNAs (Guan *et al.*, 2011; Helvik *et al.*, 2007; Jiang *et al.*, 2007; Yan *et al.*, 2007; Zhou *et al.*, 2007). Adaboost on tree kept a high score level in this study, but is always lower than forest of random trees. Compared to these classifiers, the SVM has the limit of the training time, where several hours are needed to train the model. This is a problem to try the classifier with diverse parameters, and would be a disadvantage to the usage of MiRdup*. Perhaps several tests on parameters could improve its overall results.

We know that relying only on miRBase could limit the discovery of novel miRNAs. However, we can assume that this database contains many examples of what characterizes a miRNA on its pre-miRNA. Machine learning algorithms are very useful for this kind of problem. They can accept certain diversity around usual characteristics in order to assess new members that fulfill the majority of the statistics. Also, the majority of hairpins in miRBase are single loop hairpins, but several hundred contain multiloops. We designed this algorithm so that we can accept multiloop hairpins, since we only need to focus on the duplex containing the miRNA and its complement. Consequently, if plants contain more miRNAs with multiloop pre-MiRNAs, it won't affect MiRdup*.

We noticed in this study that general statistics on features change between set of species, especially between different groups of plants. Those differences denote that specific datasets should be used depending on the hairpin predictions we want to validate. The objective of MiRdup* here is to reduce a noise as post treatment of predicted hairpins.

Additional Information references

Breiman, L. (2001) Random forests. *Machine learning* 45, 5–32.

Carollo, V., Matthews, D.E., Lazo, G.R., Blake, T.K., Hummel, D.D., Lui, N., Hane, D.L. and Anderson, O.D. (2005) Grain Genes 20 an improved resource for the small-grains community. *Plant Physiol.* 139, 643–651.

Childs, K.L., Hamilton, J.P., Zhu, W., Ly, E., Cheung, F., Wu, H., Rabinowicz, P.D., Town, C.D., Buell, C.R. and Chan, A.P. (2007) The TIGR plant transcript assemblies database. *Nucleic Acids Res.* 35, D846–D851.

Fahlgren, N., Howell, M.D., Kasschau, K.D., Chapman, E.J., Sullivan, C.M., Cumbie, J.S., Givan S.A., Law, T.F., Grant, S.R. and Dangl, J.L. (2007) High-throughput sequencing of *Arabidopsis* microRNAs: evidence for frequent birth and death of MIRNA genes. *PLoS One* 2, e219.

Freund, Y. and Schapire, R.E. (1995) A desicion-theoretic generalization of on-line learning and an application to boosting. *Computational learning theory* 904, 23–37.

Gish, W. and States, D.J. (1993) Identification of protein coding regions by database similarity search. *Nature Genet.* 3, 266–272.

Guan, D.G., Liao, J.Y., Qu, Z.H., Zhang, Y. and Qu, L.H. (2011) mirExplorer: Detecting miRNAs from genome and next generation sequencing data using the adaboost method with transition probability matrix and combined features. *RNA Biol.* 8, 922–934.

Hamel, F., Breton, C. and Houde, M. (1998) Isolation and characterization of wheat aluminum-regulated genes: possible involvement of aluminum as a pathogenesis response regulator. *Planta* 205, 531–538.

Houde, M., Belcaid, M., Ouellet, F., Danyluk, J., Monroy, A.F., Dryanova, A., Gulick, P., Bergeron, A., Laroche, A., Links, M.G., MacCarthy, L., Crosby, W.L. and Sarhan, F. (2006) Wheat EST resources for functional genomics of abiotic stress. *BMC Genomics* 7, 149.

Houde, M. and Diallo, A.O. (2008) Identification of genes and pathways associated with aluminum stress and tolerance using transcriptome profiling of wheat near-isogenic lines. *BMC Genomics* 9, 400.

Helvik,S.A., Snove, O. and Saetrom, P. (2007) Reliable prediction of drosha processing sites improves miRNA gene prediction. *Bioinformatics* 23, 142–149.

Hong, S.J. (1997) Use of contextual information for feature ranking and discretization. *IEEE Trans. Knowledge Data Engrg.* 9, 718–730.

Jiang, P., Wu, H., Wang, W., Ma, W., Sun, X. and Lu, Z. (2007) MiPred: classification of real and pseudo microRNA precursors using random forest prediction model with combined features. *Nucleic Acids Res.* 35, W339–W344.

Osman, I.H. and Kelly, J.P*.* (1996) Meta-heuristics: Theory and Applications. Kluwer Academic publisher, Norwell, Boston, MA, USA.

Quinlan, J.R. *(*1993) C4.5: Programs for Machine Learning*.* Morgan Kaufmann, Los Altos, California.
