# Supplementary material for: An integrative approach to identify hexaploid wheat miRNAome associated with development and tolerance to abiotic stress
Source: BMC Genomics. 2015 Apr 24;16(1):339. doi: 10.1186/s12864-015-1490-8 (PMC4443513; doi:10.1186/s12864-015-1490-8)
Supplement: Additional file 2: — Supplementary Tables from S1 to S11; Provides supplementary Tables of the manuscript and their associated legends. Long Tables are given xls files embedded in the zip but their legends remain in the main supplementary table files named < Tables S1to5 and S8to11.docx >. [file 12864_2015_1490_MOESM2_ESM.zip › Additional file 2/Tables S1to5 and S8to11.docx]

# Additional file 2.Contains a**ll supplementarytables and their legends. Larger tables are given in secondary files.**

**Table S1**: Description of the 10 libraries (L1 to L10). The library number corresponds to the number of the barcode used in the cDNA construction. Different tissues including aerial parts (A) which comprise leaves and crowns, spikes (Sp) and roots (Rt) from tolerant (T) or sensitive (S) genotypes in vegetative (V) or reproductive (R) phase under normal conditions (Nc) or subjected to short exposure to cold (C), long exposure to cold (vernalization; V), salt (Sa) or Al (Al). The tolerant genotypes are Clair and Atlas for cold and Al, respectively and the sensitive genotype is Bounty for both stresses. For more details, see Additional file 1: Method S1.

| **Genotype** | | **Phases** | | **Tissues** | | | **Abiotic stress** | | | | **Growth conditions and sampled time points** |  |
| --- | --- | --- | --- | --- | --- | --- | --- | --- | --- | --- | --- | --- |
| T | S | V | R | A | Rt | Sp | C/ V | Sa | Al | Nc |  |  |
| **L1** |  |  |  |  |  |  |  |  |  |  |  | Normal conditions (Nc): one, two, and three week-old plants grown at 20°C with normal watering |
| **L2** |  |  |  |  |  |  |  |  |  |  |  | Vernalization (V): two week-old plants acclimated at 4°C for 1, 2, 7, 14, 21, 28, 35, 49, 56 days |
| **L3** |  |  |  |  |  |  |  |  |  |  |  | Normal conditions: 56 day vernalized plants transferred to 20°C for 3-6 weeks |
| **L4** |  |  |  |  |  |  |  |  |  |  |  | Salt (Sa): three week-old plants of the genotype Clair watered with 200 mM of NaCl solution for 1, 3, 5, 7, 12 and 15 days |
| **L5** |  |  |  |  |  |  |  |  |  |  |  |  |
| **L6** |  |  |  |  |  |  |  |  |  |  |  | Normal conditions (Nc): 1, 2, and 3 week old plants grown at 20°C with normal watering |
| **L7** |  |  |  |  |  |  |  |  |  |  |  | Cold (C): two week old plants acclimated at 4°C for 7, 14, 21, 28 days |
| **L8** |  |  |  |  |  |  |  |  |  |  |  | Al (Al): one week old seedlings exposed to 5 µM Al for 1 day |
| **L9** |  |  |  |  |  |  |  |  |  |  |  | Normal conditions (Nc): root tissue of 1 week-old seedlings with normal watering |
| **L10** |  |  |  |  |  |  |  |  |  |  |  | Al (Al): one week-old seedlings exposed to 50 µM Al for 1 day |

**Table S2:** Quality values (QV) of predicted miRNAs color reads based on analyses of the quality files provided by SOLID sequencing in the first 10 color bases. QV<10 is the number of bases having a quality value (QV) strictly lower than 10 in the first 10 bases. First column contains the number of possible bases with quality value strictly inferior to 10 that is considered as high sequence quality. Results are shown for the ten sequenced libraries**.** The ten columns (L1-L10) contain the percentage of reads sequences in each library having quality value in bases inferior to 10; the last column corresponds to the mean in all libraries.

For detailed information about the libraries and conditions seeAdditional file 1: Method S1 and Additional file 2: Table S1.

| QV<10 | L1 | L2 | L3 | L4 | L5 | L6 | L7 | L8 | L9 | L10 | Mean |
| --- | --- | --- | --- | --- | --- | --- | --- | --- | --- | --- | --- |
| 0 | 95,73 | 94,74 | 96,03 | 94,20 | 94,72 | 94,46 | 93,36 | 95,22 | 95,78 | 94,11 | 94,74 |
| 1 | 0,57 | 0,63 | 0,47 | 0,74 | 0,52 | 0,64 | 0,73 | 0,61 | 0,70 | 0,80 | 0,64 |
| 2 | 0,52 | 0,62 | 0,40 | 0,70 | 0,60 | 0,60 | 0,72 | 0,63 | 0,58 | 0,71 | 0,61 |
| 3 | 0,44 | 0,54 | 0,34 | 0,60 | 0,46 | 0,55 | 0,67 | 0,47 | 0,42 | 0,63 | 0,53 |
| 4 | 0,46 | 0,52 | 0,39 | 0,70 | 0,53 | 0,54 | 0,62 | 0,51 | 0,44 | 0,58 | 0,54 |
| 5 | 0,39 | 0,51 | 0,35 | 0,59 | 0,49 | 0,54 | 0,66 | 0,50 | 0,39 | 0,55 | 0,51 |
| 6 | 0,38 | 0,50 | 0,41 | 0,50 | 0,46 | 0,53 | 0,64 | 0,47 | 0,38 | 0,49 | 0,49 |
| 7 | 0,37 | 0,46 | 0,41 | 0,53 | 0,49 | 0,51 | 0,63 | 0,46 | 0,33 | 0,51 | 0,48 |
| 8 | 0,38 | 0,48 | 0,40 | 0,49 | 0,56 | 0,54 | 0,66 | 0,42 | 0,34 | 0,54 | 0,49 |
| 9 | 0,38 | 0,48 | 0,35 | 0,45 | 0,54 | 0,54 | 0,65 | 0,35 | 0,32 | 0,50 | 0,47 |
| 10 | 0,37 | 0,52 | 0,45 | 0,50 | 0,63 | 0,54 | 0,67 | 0,35 | 0,33 | 0,58 | 0,50 |

**Table S3:**The 35 miRNA features used by MiRdup* to classify pre-miRNA candidates and their importance in the whole prediction. Features ranking on all sequenced or cloned miRNAs from miRBase dataset with the ranker of InfoGain. For each instance containing the miRNA, pre-miRNA and the secondary structure, 35 features are extracted. IDs were set arbitrarily. Gray area represents the 14 best features having a rank superior to 0.05.

| Rank score | ID | Features |
| --- | --- | --- |
| 0.27457256 | 22 | Distance from terminal loop |
| 0.22249313 | 25 | Length of overlap in loop |
| 0.19002714 | 28 | Average number of paired bases in window 3 |
| 0.18625003 | 35 | Length of biggest bulges in percentage of the miRNA length |
| 0.18453715 | 34 | Length of biggest bulge |
| 0.17837146 | 27 | Average number of paired bases in window 5 |
| 0.16974265 | 7 | Number of base pairs in duplex |
| 0.16260705 | 26 | Average number of paired bases in window 7 |
| 0.15725944 | 2 | Duplex minimum folding energy |
| 0.11455883 | 24 | miRNA included in loop |
| 0.07013466 | 23 | Distance from hairpin start |
| 0.0648666 | 13 | Start of perfect 5 mer base pair |
| 0.05432927 | 5 | Maximum length without bulges in percentage of the miRNA length |
| 0.05422537 | 4 | Maximum length without bulges |
| 0.0262861 | 11 | Start of perfect 10 mer base pair |
| 0.02460451 | 6 | Length without bulges from miRNA start |
| 0.02011416 | 12 | Presence of perfect 5 mer base pair |
| 0.01970081 | 10 | Presence of perfect 10 mer base pair |
| 0.01560982 | 29 | Bulge at position 2 |
| 0.01496887 | 3 | GC percentage |
| 0.01059411 | 30 | Bulge at position minus 2 |
| 0.00826505 | 32 | Bulge at position minus 1 |
| 0.00707627 | 20 | Percentage of G in the miRNA |
| 0.00453195 | 9 | Start of perfect 20 mer base pair |
| 0.00435343 | 8 | Presence of perfect 20 mer base pair |
| 0.00402892 | 33 | Number of bulges |
| 0.00363068 | 18 | Percentage of A in the miRNA |
| 0.0029013 | 19 | Percentage of U in the miRNA |
| 0.00164901 | 21 | Percentage of C in the miRNA |
| 0.001163 | 17 | Presence of C |
| 0.00042975 | 31 | Bulge at position 1 |
| 0.00007034 | 16 | Presence of G |
| 0.00002714 | 14 | Presence of A |
| 0.00000199 | 15 | Presence of U |
| 0 | 1 | miRNA length |

**Table S4:** Quality values (QV) of predicted miRNAs color reads (corresponding to miRNA candidates) based on analyses of the quality files provided by SOLID sequencing in the first 10 color bases. QV<10 is the number of bases having a quality value (QV) strictly lower than 10 in the first 10 bases. Since one predicted miRNA can come from many reads, we calculate the average QV<10 and round it down to integer. First column contains the number of possible bases with quality value strictly inferior to 10 that is considered as high sequence quality. The second column is the number of reads of the deep sequencing. The third column is the corresponding percentage of mapping. The fourth column is the number of predicted miRNAs. The fifth column is the percentage of predicted miRNAs compared to 1369. The sixth column is the number of reads (several reads can have the same sequence) and the seventh column is the corresponding percentage. For detailed information about the libraries and conditions see Additional file 1: Method S1 and Additional file 2: Table S1.

.

| **Bases with QV<10** | **Number**  **of reads** | **% of mapping** | **Predicted miRNAs** | **% of predicted miRNAs** | **Reads** | **% of reads** |
| --- | --- | --- | --- | --- | --- | --- |
| 0 | 50031306 | 56,15 | 128 | 64,32 | 833611 | 94,82 |
| 1 | 13518710 | 15,17 | 4 | 2,01 | 5556 | 0,63 |
| 2 | 9281699 | 10,42 | 5 | 2,51 | 5323 | 0,61 |
| 3 | 5812834 | 6,52 | 5 | 2,51 | 4589 | 0,52 |
| 4 | 4189907 | 4,70 | 3 | 1,51 | 4694 | 0,53 |
| 5 | 2426248 | 2,72 | 4 | 2,01 | 4424 | 0,50 |
| 6 | 1641104 | 1,84 | 3 | 1,51 | 4216 | 0,48 |
| 7 | 915577 | 1,03 | 6 | 3,02 | 4133 | 0,47 |
| 8 | 862935 | 0,97 | 7 | 3,52 | 4206 | 0,48 |
| 9 | 273630 | 0,31 | 7 | 3,52 | 4076 | 0,46 |
| 10 | 151146 | 0,17 | 27 | 13,57 | 4343 | 0,49 |

**Table S5:** The different explored thresholds (Evalue) and Query/Hit coverage and percentage identity of ESTs producing the identified pre-miRNAs aligned (blasted) against a) TREP database for transposable elements; and b) plant proteins database. Results of blast are presented. For the remaining analysis, we retained 1.00E-20 and Query Coverage (QC) or Hit Coverage (HC) ≥ 85; and percentage of identity ≥75 for proteins.

**a)**

| **Evalue ≤** | **percent identity ≥** | **HC ≥** | **Pre-miRNAs overlaping TE** | **miRNAs overlaping TE** |
| --- | --- | --- | --- | --- |
| **0.1** | 80 | 0 | 136 | 29 |
| **0.00005** | 80 | 85 | 74 | 13 |
| **0.00005** | 95 | 85 | 31 | 13 |
| **1.00E-20** | 80 | 0 | 97 | 0 |
| **1.00E-20** | 80 | 85 | 68 | 0 |
| **1.00E-20** | 95 | 85 | 31 | 0 |
| **EV ≤ 0,00005 and EV ≥ 1E-20** | 80 | 0 | 116 | 13 |

**b)**

| Evalue ≤ | QC or HC ≥ | percentage identity ≥ | ESTs aligned with protein | ESTs producing miRNAs overlapping proteins | Number of miRNA overlapping proteins |
| --- | --- | --- | --- | --- | --- |
| ∞ | 0 | 0 | 320 | 171 | 28 |
| 5,00E-05 | 0 | 0 | 308 | 163 | 27 |
| 5,00E-05 | 85 | 75 | 77 | 54 | 9 |
| 1,00E-20 | 0 | 0 | 232 | 148 | 20 |
| 1,00E-20 | 85 | 75 | 76 | 54 | 9 |
| EV ≤ 0,00005 and EV ≥ 1E-20 | 0 | 0 | 221 | 77 | 22 |

**Table S6**: List of predicted target genes and their associated Uniref and GO Slimterms when available. See excel file ***S7_TargetGenes.xlsx***.

**Table S7**: Enrichment of GO Slim terms in the three gene ontology categories (*cell component*, *molecular function* and *biological process*) for targets of all miRNAs predicted from the ten sequenced libraries. See excel file ***S8_GeneOntolgyEnrichmentAnalysis.xlsx.***

**Table S8**: The number of miRNA abundance level per library (low, 10-99 reads; medium, 100-999 reads; and high, 1000 and more) and the percentage of the overall abundance of the given miRNA and others small RNAs mapped or positioned in a given pre-miRNA. The percentage of abundance of miRNA candidates is partitioned in three groups (low, medium, high) indicating the percentage between respectively [100..51], [50..31] and [30..0] of a given pre-miRNA. For detailed information about the libraries and conditions see Additional file 2: Table S1.

.

| **L1** |  |  |  |  | **L2** |  |  |  |
| --- | --- | --- | --- | --- | --- | --- | --- | --- |
| **Level** | **0-30** | **31-50** | **51-100** |  | **Level** | **0-30** | **31-50** | **51-100** |
| Low | 9 | 7 | 54 |  | Low | 7 | 12 | 60 |
| Medium | 3 | 9 | 52 |  | Medium | 8 | 9 | 67 |
| High | 0 | 1 | 16 |  | High | 0 | 2 | 23 |
|  | 12 | 17 | 122 |  |  | 15 | 23 | 150 |
| **L3** |  |  |  |  | **L4** |  |  |  |
| **Level** | **0-30** | **31-50** | **51-100** |  | **Level** | **0-30** | **31-50** | **51-100** |
| Low | 5 | 9 | 63 |  | Low | 9 | 14 | 77 |
| Medium | 1 | 2 | 22 |  | Medium | 2 | 6 | 46 |
| High | 0 | 0 | 6 |  | High | 0 | 0 | 16 |
|  | 6 | 11 | 91 |  |  | 11 | 20 | 139 |
| **L5** |  |  |  |  | **L6** |  |  |  |
| **Level** | **0-30** | **31-50** | **51-100** |  | **Level** | **0-30** | **31-50** | **51-100** |
| Low | 7 | 10 | 78 |  | Low | 6 | 7 | 52 |
| Medium | 6 | 9 | 43 |  | Medium | 2 | 7 | 50 |
| High | 0 | 0 | 7 |  | High | 0 | 2 | 16 |
|  | 13 | 19 | 128 |  |  | 8 | 16 | 118 |
| **L7** |  |  |  |  | **L8** |  |  |  |
| **Level** | **0-30** | **31-50** | **51-100** |  | **Level** | **0-30** | **31-50** | **51-100** |
| Low | 9 | 8 | 66 |  | Low | 9 | 8 | 67 |
| Medium | 2 | 9 | 43 |  | Medium | 4 | 10 | 50 |
| High | 0 | 1 | 14 |  | High | 1 | 0 | 8 |
|  | 11 | 18 | 123 |  |  | 14 | 18 | 125 |
| **L9** |  |  |  |  | **L10** |  |  |  |
| **Level** | **0-30** | **31-50** | **51-100** |  | **Level** | **0-30** | **31-50** | **51-100** |
| Low | 7 | 12 | 73 |  | Low | 8 | 9 | 67 |
| Medium | 6 | 7 | 39 |  | Medium | 4 | 8 | 44 |
| High | 1 | 0 | 5 |  | High | 2 | 0 | 7 |
|  | 14 | 19 | 117 |  |  | 14 | 17 | 118 |

**Table S9**: Number and characteristics of differentially expressed miRNAs under different growth conditions. Normalized reads were compared between normal conditions and treatments (cold, salt and Al) for a given genotype to identify miRNAs associated with stress responses and between tolerant (winter wheat) and sensitive (spring wheat) genotypes to identify miRNAs associated with tolerance. For development, normalized reads were compared between plants in vegetative and reproductive phases. MiRNAs showing a fold change (FC) of two or more with an adjusted p-value ≤ 0.05 in a given condition are presented. MiRNAs regulated in response to vernalization (L2/L1) and salt (L4/L1) in winter wheat, cv Clair; cold (L7/L6) in spring wheat, cv Bounty; Al (L8/L9) in sensitive spring, cv Bounty and (L10/L9) in tolerant winter wheat, cv Atlas and their respective fold changes. For Al treatment, the library control from spring wheat (Bounty) was not sequenced; we used the control library from winter wheat (L9) for differential miRNAs expression analysis since our results from previous studies showed that the 2 genotypes share high similarity in their basal mRNAs expression (Method S1). MiRNAs associated with tolerance shownin this Table are only those identified by comparing 2 treated libraries, L2/L7 for cold and L10/L8 for Al, see Table S11 for more information on miRNAs associated with tolerance. Development responsive miRNAs were identified by comparing: first, vernalized plants during vegetative phase and de-acclimated plants (vernalized plants transferred to normal conditions) during the reproductive phase (L3/L2). This corresponds to miRNAs associated with floral transition; second, between control plants (un-vernalized) during vegetative phase and de-acclimated plants during reproductive phase (L3/L1). This corresponds to miRNAs associated with flowering. For detailed information see Additional file 1: Method S1 and Additional file 2: Table S1.

.

|  | Conditions | Libraries | Number of expressed miRNAs | Number of differentially expressed miRNAs |  | Maximum FC up | Maximum FC down |
| --- | --- | --- | --- | --- | --- | --- | --- |
| Abiotic stress responses | Vernalization  (winter wheat) | L2/L1 | 199 | 67 |  | 72 | 25 |
|  | Cold  (spring wheat) | L7/L6 | 198 | 34 |  | 17 | 7 |
|  | Al (winter wheat) | L10/L9 | 192 | 85 |  | 23 | 39 |
|  | Al (spring wheat | L8/L9 | 191 | 86 |  | 14 | 30 |
|  | Salt in leaves (winter wheat cv Clair) | L4/L1 | 198 | 55 |  | 82 | 41 |
|  | Total unique abiotic stress responsive miRNAs |  | - | **165** |  | - | - |
| Tolerance | Cold tolerance | L2/L7 | 199 | 52 |  | 17 | 21 |
|  | Al tolerance | L10/L08 | 190 | 27 |  | 4 | 23 |
|  | Total unique miRNAs |  | - | **69** |  | - | - |
| Development responses | Floral transition | L3/L2 | 199 | 76 |  | 18 | 67 |
|  | Flowering | L3/L1 | 194 | 56 |  | 63 | 17 |
|  | Total unique development responsive miRNAs | - | - | **99** |  | - | - |
|  | Total unique |  | **199** | **182** |  | - | - |

**Table S10: Grouping miRNAs based on their digital gene expression patterns.** Expressions of cold and Al stress responsive miRNAs were compared between spring and winter wheats (sensitive/tolerant). Expression of development responsive miRNAs was compared under different developmental stages (vegetative /reproductive). Abiotic stress and developmentally responsive miRNAs were grouped into 24 groups (eight per investigated condition). The groups Co1-Co8 include miRNAs associated with cold/vernalization responses. They show similar (Co5, Co6) or different (CO1-4 and CO7-8) expression patterns in response to long exposure to cold (4^o^C up to 56 days) in winter wheat (cv Clair, the tolerant genotype) (L2/L1) compared to short exposure to cold (at 4^o^C up to 28 days) in spring wheat (cv Bounty, the sensitive genotype) (L7/L6), during vegetative phase. The groups Al1-Al8 include miRNAs associated with Al responses. They show similar (Al5, Al6) or different (Al1-4 and Al7-8) expression patterns in response to Al in winter wheat (cv Atlas, the tolerant genotype) (L10/L9) compared to spring wheat (cv Bounty, the sensitive genotype) (L8/L9) at the same development stage. The groups showing different expression patterns between the sensitive and the tolerant genotype are associated with cold (CO1-4 and CO7-8) and Al (Al1-4 and Al7-8) tolerance. The groups 5 (Co5, Al5) and 6 (Co6, Al6) are associated with cold and Al responses, respectively.The control library from spring wheat (Bounty) for Al treatment was not sequenced. We used the control library from winter wheat (L9) for differential miRNAs expression analysis since our results from previous studies showed that the 2 genotypes share high similarity in their basal mRNAs expression (Method S1). The groups Dev1-Dev8 include miRNAs associated with plant development in winter wheat cv Clair. They are differentially expressed between vernalized plants(cold acclimated winter wheat plants at 4^o^C up to 56 days)during the vegetative phase and de-acclimated plants (vernalized plants transferred to normal conditions) during the reproductive phase (L3/L2); and between control plants (un-vernalized) during the vegetative phase and de-acclimated plants during the reproductive phase (L3/L1). dw, down-regulated miRNA; up, up-regulated miRNA; not, not differentially expressed. L2/L1, vernalized library L2 vs control library L1 in winter wheat (Clair); L7/L6, cold acclimated library L7 vs control library L6 in spring wheat (Bounty); L10/L9, Al library L10 vs control library L9 in winter wheat (Atlas); L8/L9, Al library L8 in spring wheat (Bounty) vs control library L9 in winter wheat (Atlas). L3/L2, reproductive library L3 vs vernalized library L2 at vegetative phase; L3/L1, library at reproductive phase L3 vs control library L1 at vegetative phase. For detailed information about the libraries and conditions seeAdditional file 1: Method S1 and Additional file 2: Table S1.

| **Investigated conditions and tissues types** | **MiRNA groups** | **Expression patterns** | **Number of miRNAs in each group** |
| --- | --- | --- | --- |
| **Cold/vernalization**  **(Aerial parts)** | **Cold tolerance** |  |  |
|  | **Co1** | not_L2/L1_up_L7/L6 | 11 |
|  | **Co2** | not_L2/L1_dw_L7/L6 | 12 |
|  | **Co3** | up_L2/L1_not_L7/L6 | 39 |
|  | **Co4** | dw_L2/L1_not_L7/L6 | 17 |
|  | **Co7** | up_L2/L1_dw_L7/L6 | 1 |
|  | **Co8** | dw_L2/L1_up_L7/L6 | 1 |
|  | **Cold response** |  |  |
|  | **Co5** | up_L2/L1_up_L7/L6 | 8 |
|  | **Co6** | dw_L2/L1_dw_L7/L6 | 1 |
| **Aluminum**  **(root tips)** |  | | |
|  | **Al1** | not_L10/L9_up_L8/L9 | 19 |
|  | **Al2** | not_L10/L9_dw_L8/L9 | 5 |
|  | **Al3** | up_L10/L9_not_L8/L9 | 14 |
|  | **Al4** | dw_L10/L9_not_L8/L9 | 9 |
|  | **Al7** | up_L10/L9_dw_L8/L9 | 0 |
|  | **Al8** | dw_L10/L9_up_L8/L9 | 2 |
|  | **Al responses** |  |  |
|  | **Al5** | up_L10/L9_up_L8/L9 | 42 |
|  | **Al6** | dw_L10/L9_dw_L8/L9 | 18 |
|  |  |  |  |
| **Development**  **(vegetative and reproductive tissues)** | **Dev1** | not_L3/L2_up_L3/L1 | 11 |
|  | **Dev2** | not_L3/L2_dw_L3/L1 | 12 |
|  | **Dev3** | up_L3/L2_not_L3/L1 | 5 |
|  | **Dev4** | dw_L3/L2_not_L3/L1 | 38 |
|  | **Dev5** | up_L3/L2_up_L3/L1 | 8 |
|  | **Dev6** | dw_L3/L2_dw_L3/L1 | 20 |
|  | **Dev7** | up_L3/L2_dw_L3/L1 | 1 |
|  | **Dev8** | dw_L3/L2_up_L3/L1 | 4 |

**Table S11: Oligonucleotides used as probes in northern blot analysis.**

| MiRNAs ID | Oligonucleotide Sequence (5'-3') | | Length nt | |
| --- | --- | --- | --- | --- |
| MiRNAs predicted in common by MiRdup* and MIRcheck (CM*M) | | | | |
| apMir_20602 | | CTCCGTTCCAATATAGATGAC | | 21 |
| apMir_19980 | | GGGTGATGGATGATCGATG | | 19 |
| apMir_14769 | | TCAATACATATATGACAAC | | 19 |
| apMir_21052 | | GTATTGGGTAATCTCATCTCA | | 21 |
|  | |  | |  |
| Predicted MiRNAs specific to MiRdup* (SM*) | | | |  |
|  | |  | |  |
| apMir_16808 | | TGGTAGGATGGATGATGCTAT | | 21 |
| apMir_86202 | | ACGGGCCGCACCGCTGGCCGACCCT | | 25 |
| apMir_54471 | | GTGCCGGATTATGACTGA | | 18 |
|  | |  | |  |
| Conserved miRNAs predicted in common by MiRdup* and MIRcheck (CM*M) | | | | |
| apMir_22246 (tae-miR160a) | | TGGCATACAGGGAGCCAGGCA | | 21 |
| apMir_20968 (miR395a-21) | | AGAGTTCCCCCAAACACTTCA | | 21 |
