# Supplementary material for: An integrative approach to identify hexaploid wheat miRNAome associated with development and tolerance to abiotic stress
Source: BMC Genomics. 2015 Apr 24;16(1):339. doi: 10.1186/s12864-015-1490-8 (PMC4443513; doi:10.1186/s12864-015-1490-8)
Supplement: Additional file 3: — Supplementary Figures from S1 to S7; Provides list of supplementary figures. [file 12864_2015_1490_MOESM3_ESM.docx]

**Additional file 3.Contains the supplementary figures from Figure S1 to Figure S7**


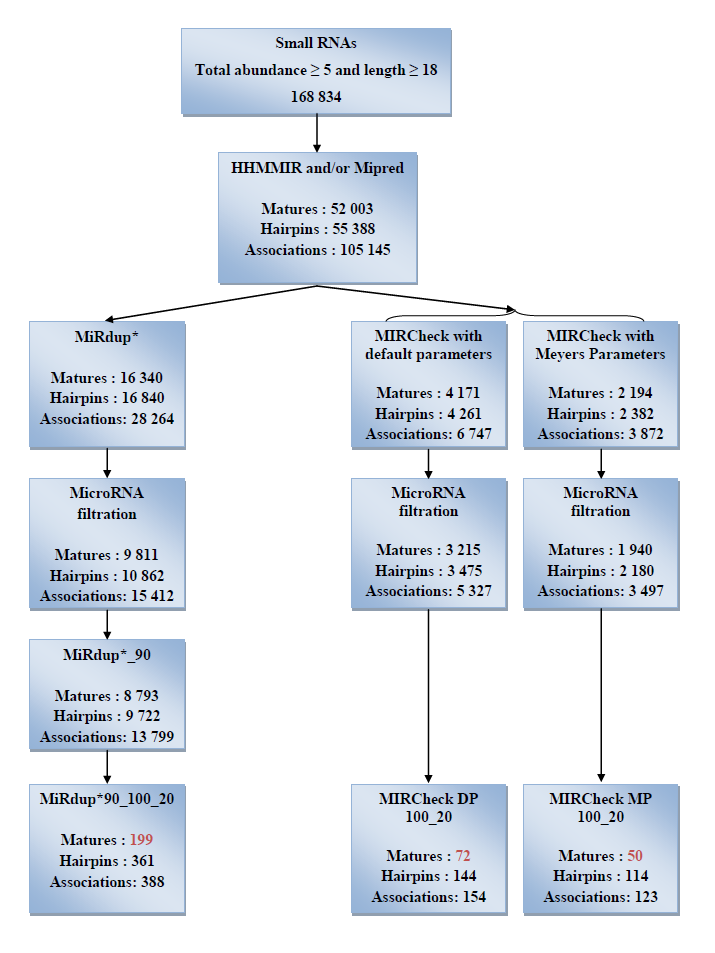


**Figure S1:** The miRNA filtering pattern comparing MiRdup* trained on all experimental miRNAs of miRBase to MIRcheck default parameters (DP) and MIRcheck with rules from Meyers *et al.,* (2008) (MP). Each bloc presents the number of mature miRNAs, hairpins and mature miRNA-Hairpin associations. MiRNAs filtration, all predicted miRNAs were filtered against Rfam to eliminate non coding RNAs (rRNA and tRNA), and against Repeat Masker to eliminate miRNAs with low complexity. MiRdup*_90, miRNAs predicted by MiRdup* with a score higher than 90. MiRdup*90_10_20, MiRdup* predicted miRNAs which satisfy two expression criteria from Meyers *et al.*, (2008): the criteria 100, represents a minimum of 100 reads in abundance in at least one sequenced library; the relaxed criteria 20 (instead 25), represents the abundance of a given miRNA represents more than 20% of the observed small RNAs in the corresponding pre-miRNA. Less than 5% of miRNAs candidates satisfy criteria ranged from 20-25%.

| **MIRcheck with default parameters** | **MIRcheck with Meyers parameters** |  |
| --- | --- | --- |
| 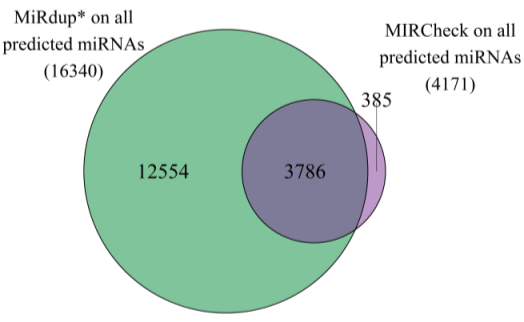 | 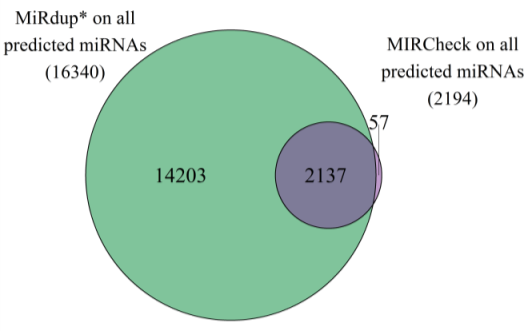 | The intersection of miRNA candidates from ***HHMMiR and MiPred*** and MIRcheck DP as well as MIRcheck MP. |
| 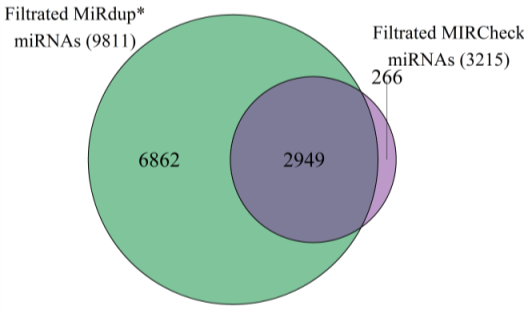 | 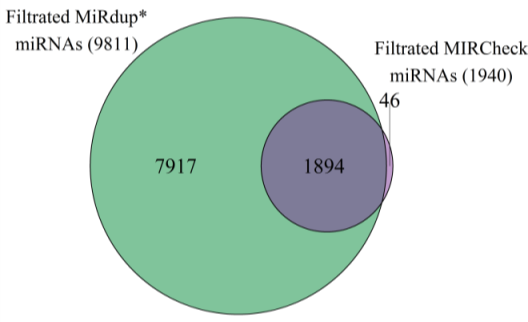 | The intersection of filtered miRNA candidates after ***MiRdup* without a threshold*** and MIRcheck DP as well as MIRcheck MP. |
| 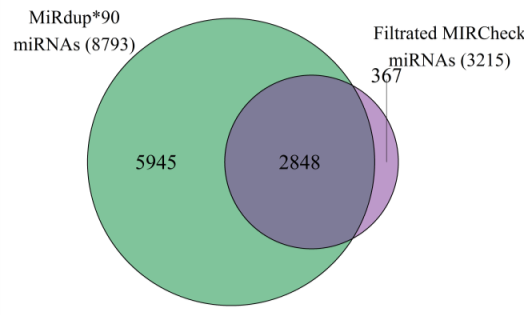 | 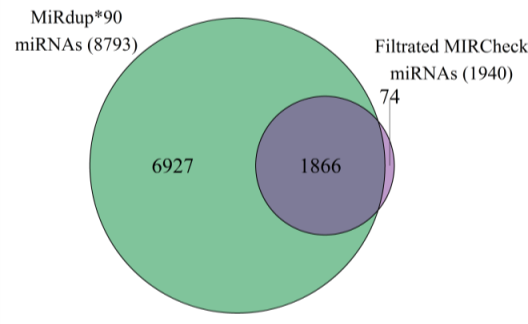 | The intersection of filtered miRNA candidates after ***MiRdup* with threshold score above 90*** and MIRcheck DP as well as MIRcheck MP. |
| 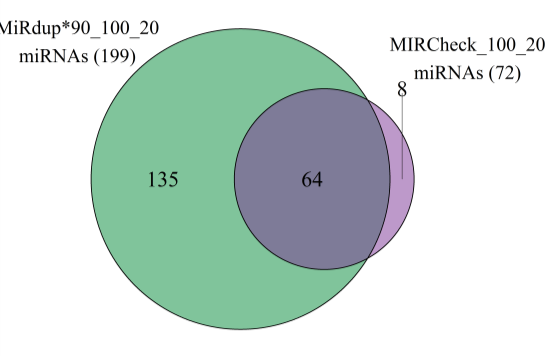 | 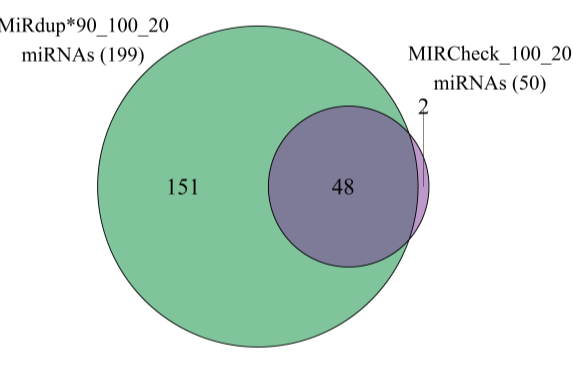 | The intersection of filtered miRNA candidates after ***MiRdup* with threshold score above 90, abundance higher than 100 reads in a library and representing above 20%*** of all small RNAs mapped to the pre-MiRNAs compared to MIRcheck DP as well as MIRcheck MP. |

**Figure S2:** The Predicted miRNAs intersection between the methods MiRdup*, MIRcheck with default parameters (DP) and MIRcheck with rules from Meyers *et al.,* (2008) (MP).

| **a**  **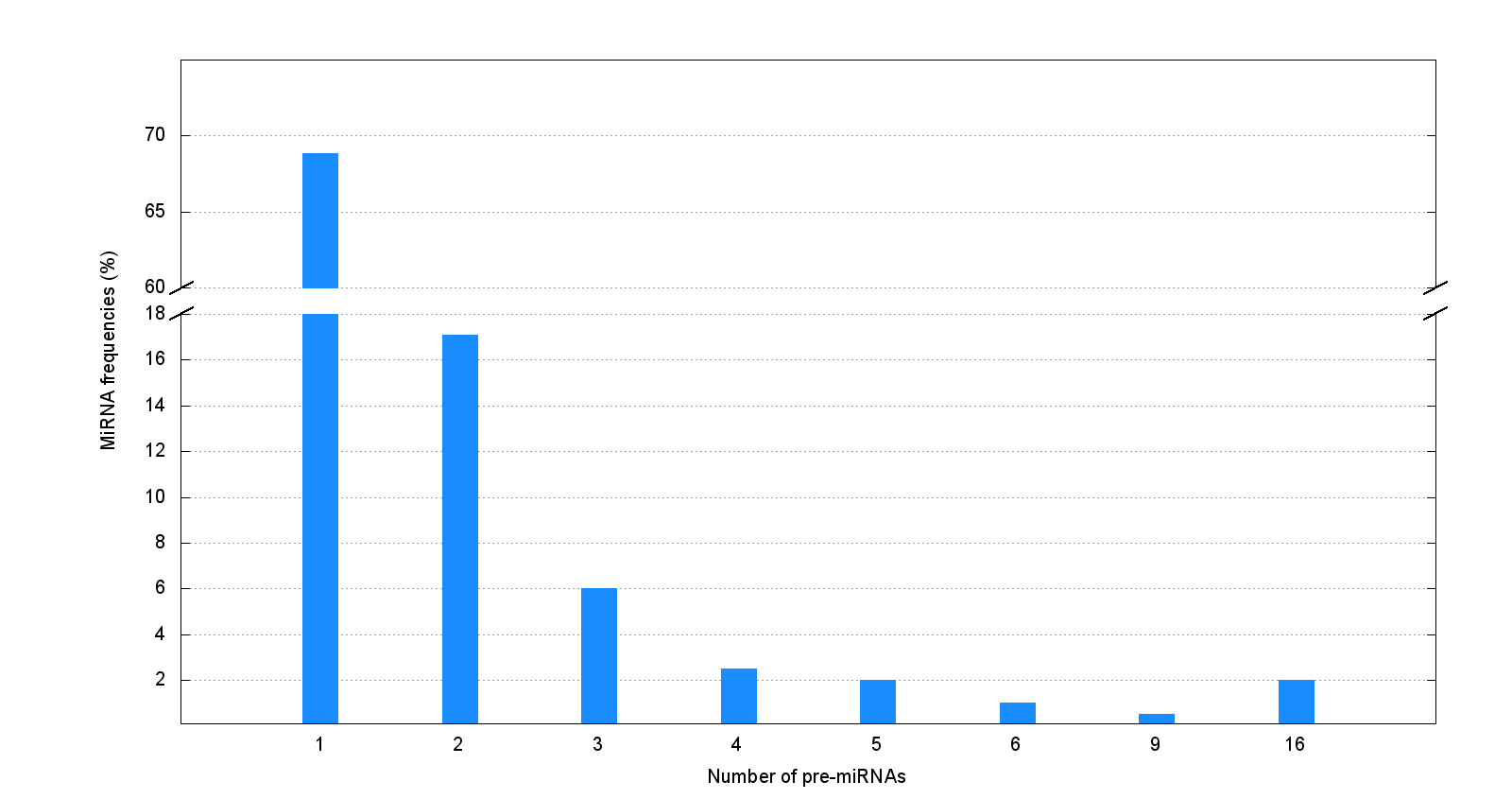** |
| --- |
| **b**  **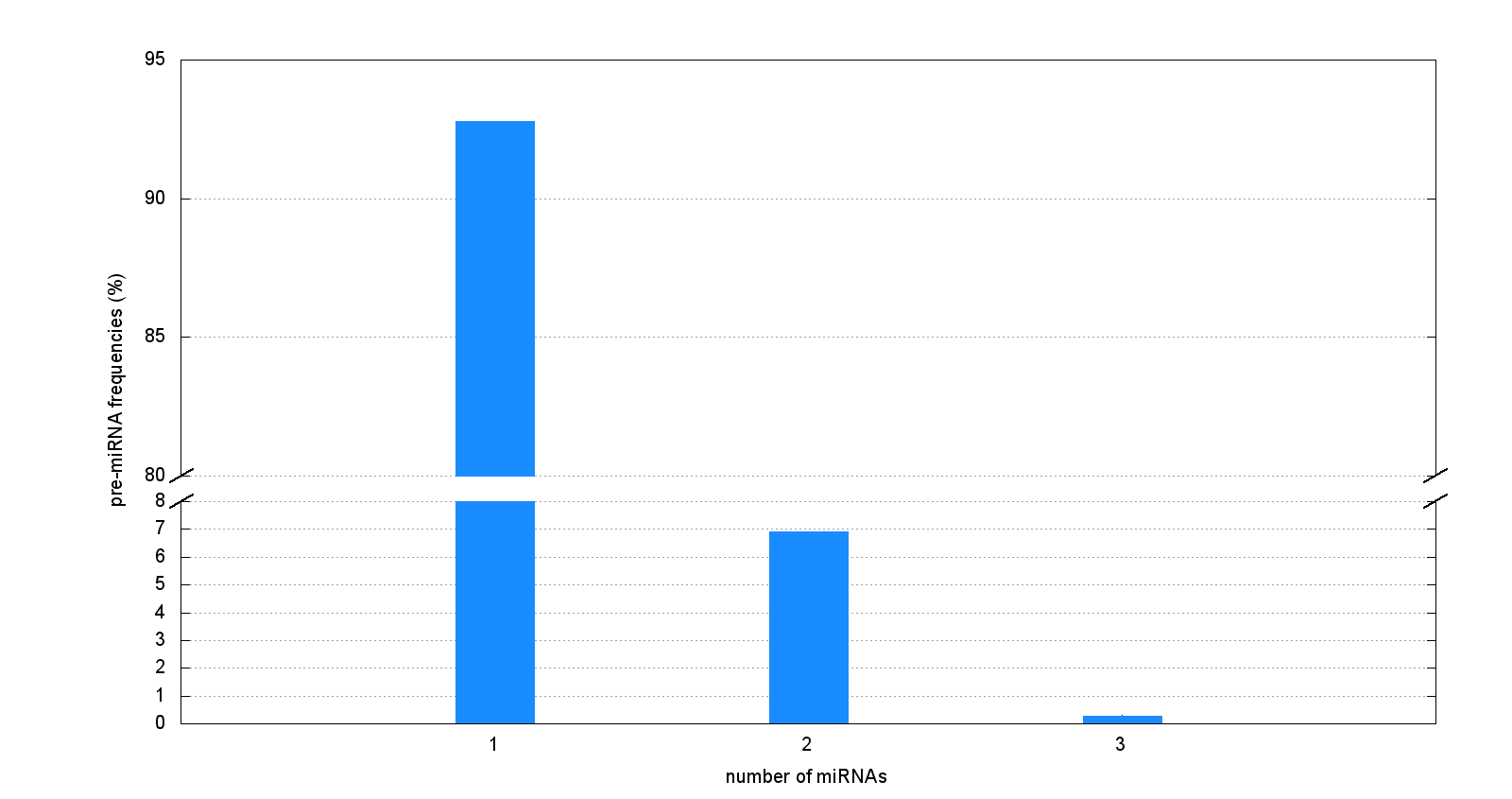** |

| **c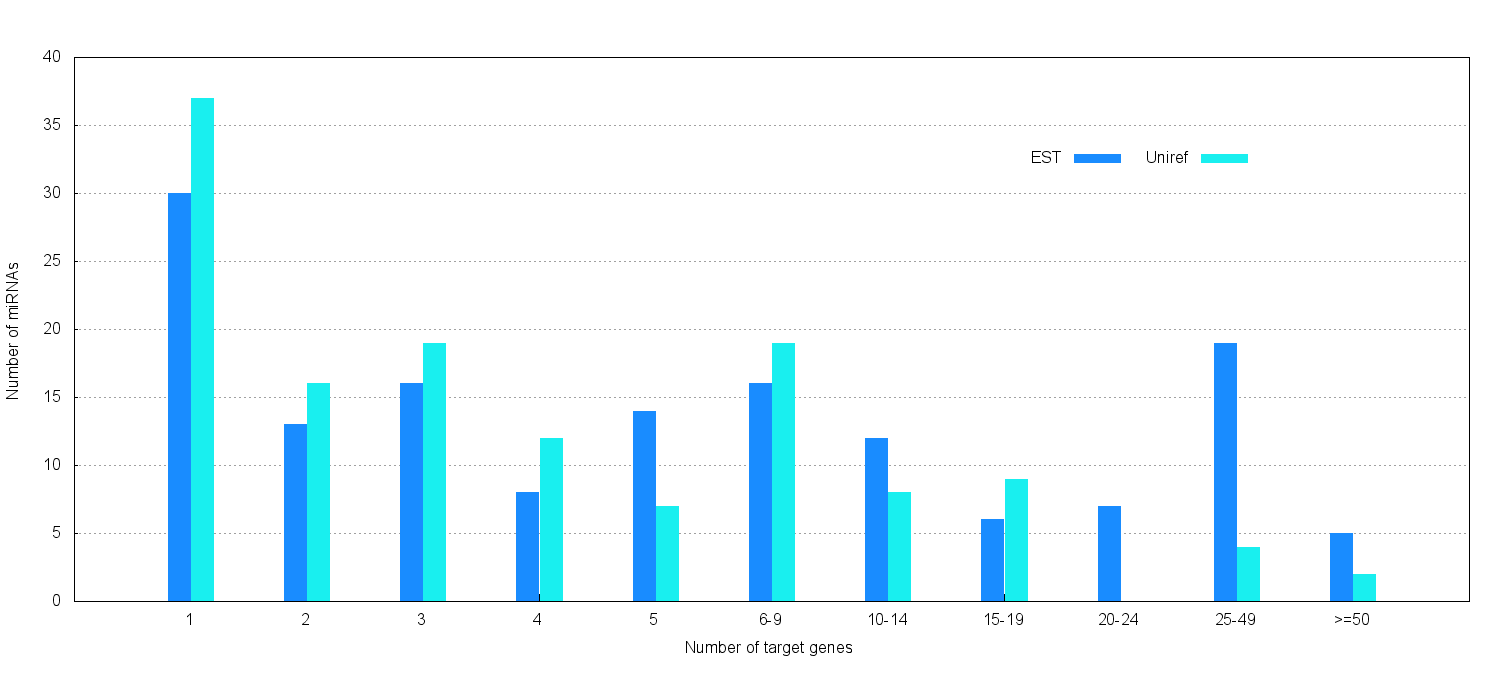** |
| --- |
| **d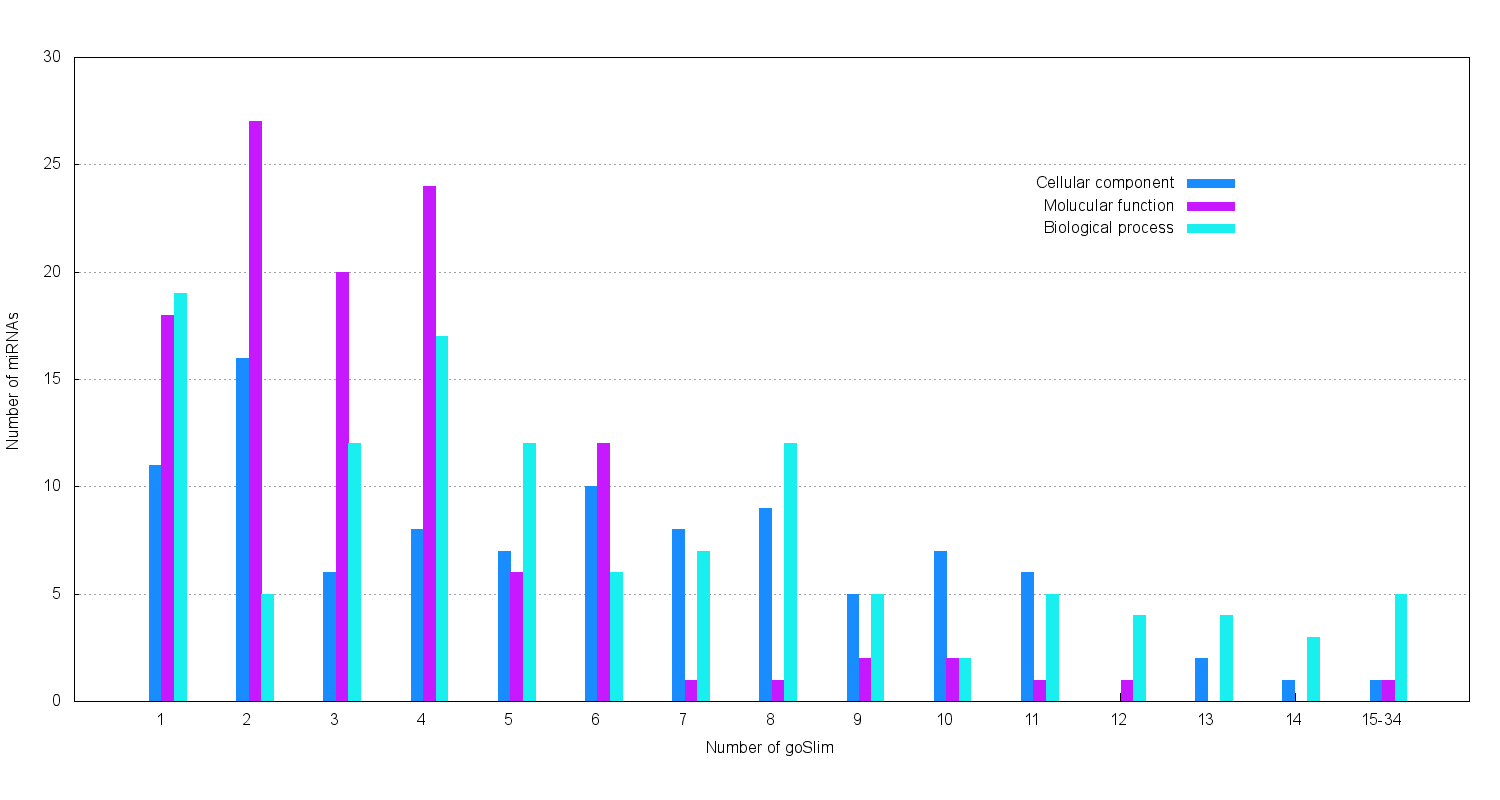** |

**Figure S3. Main characteristics of predicted miRNAs.** a) Percentage of predicted miRNAs with a given number of pre-miRNA; b) Percentage of pre-miRNAs carrying a given number of distinct predicted miRNAs; c) Fraction of predicted miRNAs targeting a given number of ESTs or UniRef ids; d) the fraction of predicted miRNAs targeting a given number of GO Slim term in the 3 main Gene Ontology categories. The number and the description of these GO Slim terms are presented in Figures S4a-S6a and Additional file 2: Table S7

| **a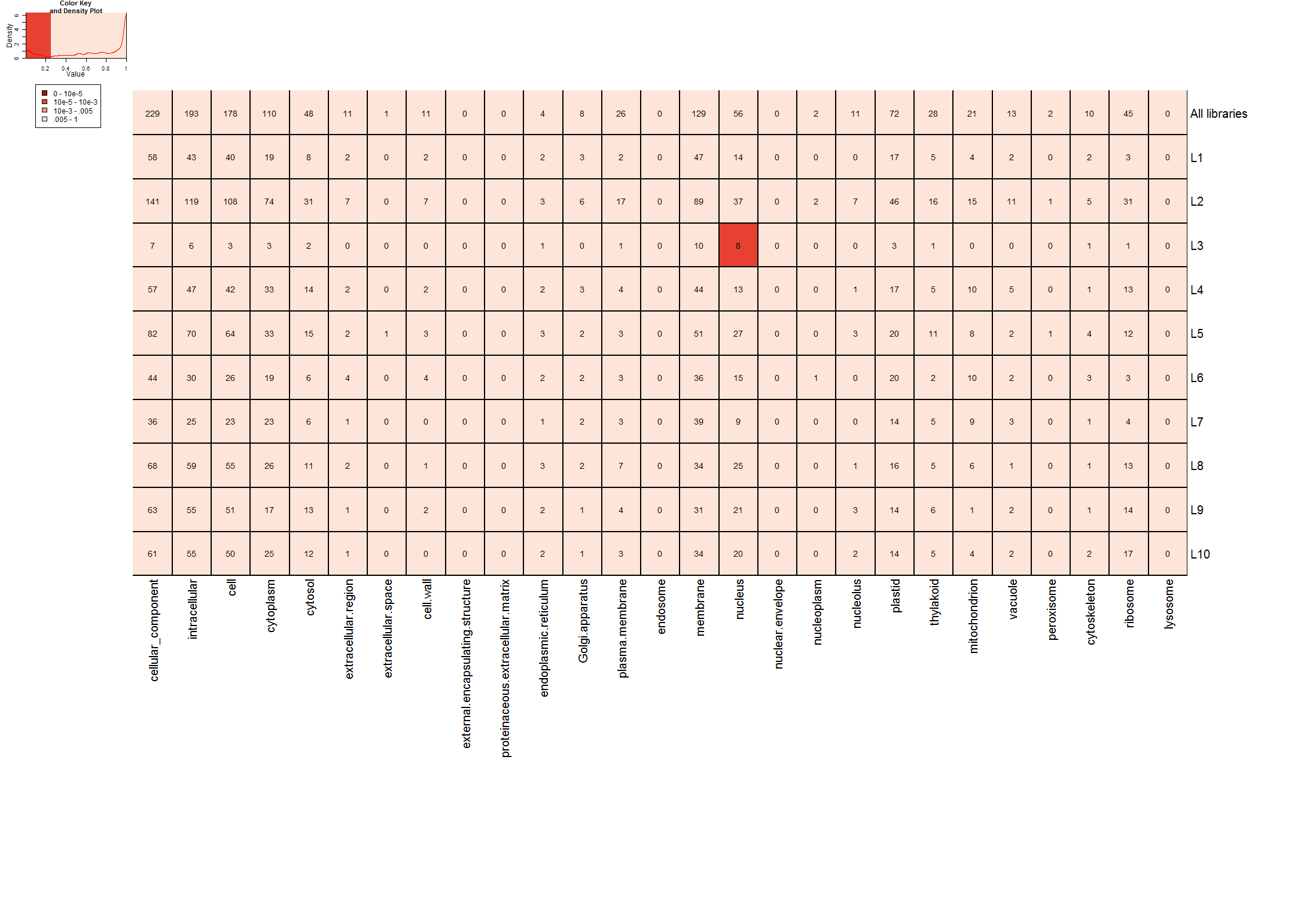** |
| --- |

| **b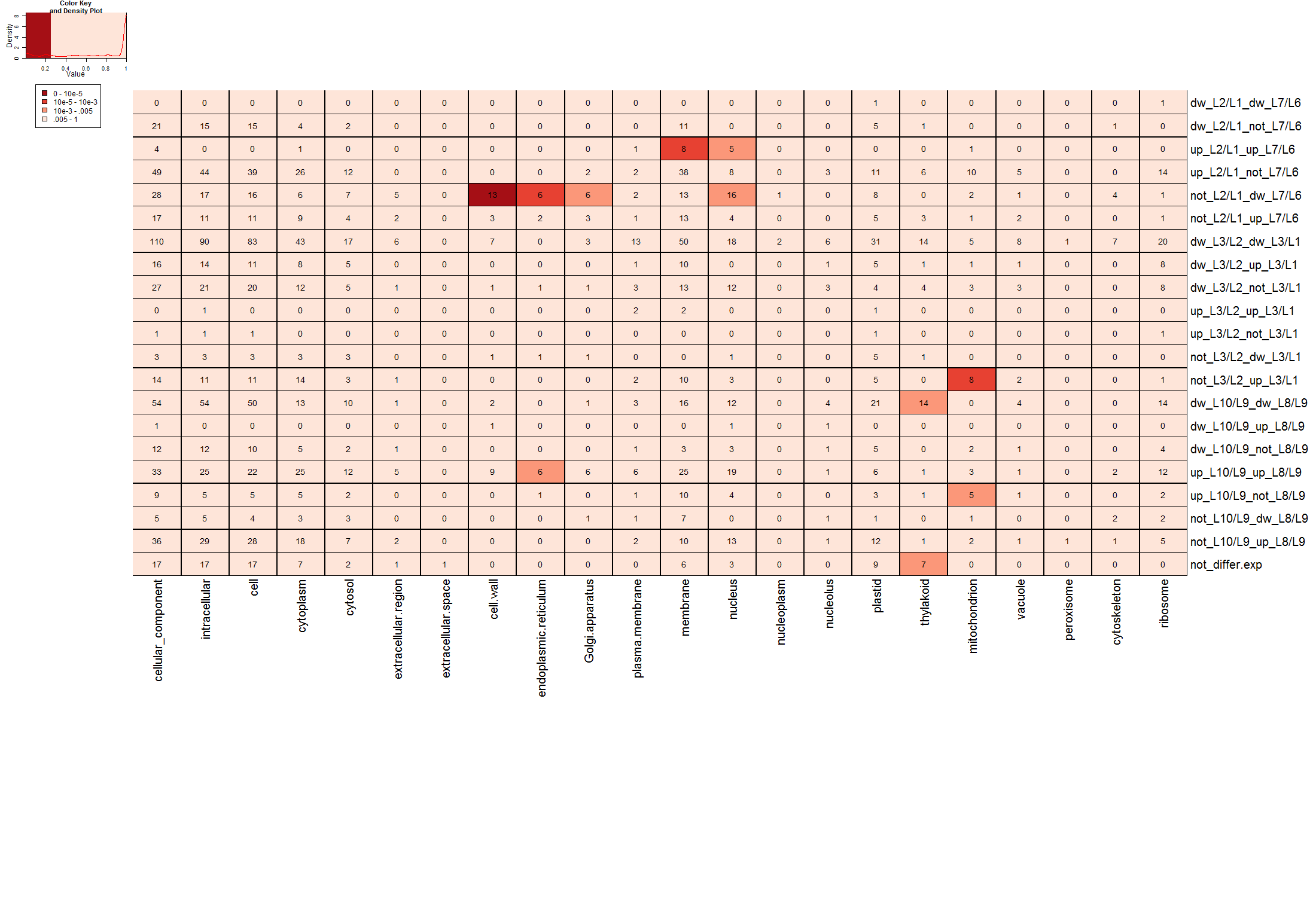** |
| --- |

**Figure S4: Enrichment of main cell component GO Slims.** a) Enrichment for the target genes in different libraries. b) Enrichment for the target genes of regulated miRNA candidates, under cold, Al, and development, grouped on 24 groups (eight per investigated condition) based on their differential expression patterns described in Additional file 2: Table S11. Dw,down regulated; up, up-regulated; not, not regulated. The value in each case indicates the number of miRNA-GO associations for the corresponding GO Slim.Targets associated with more than one cell component were assigned to more than one GO Slim term.The enrichment is presented in four different colors (high enrichment (P-value < 10^-5^), medium enrichment (P-value < 10^-3^), low enrichment (P-value < 0.05) and no enrichment (P-value ≥ 0.05). MiRNA groups having targets not annotated with a UniRef are not presented.

| **a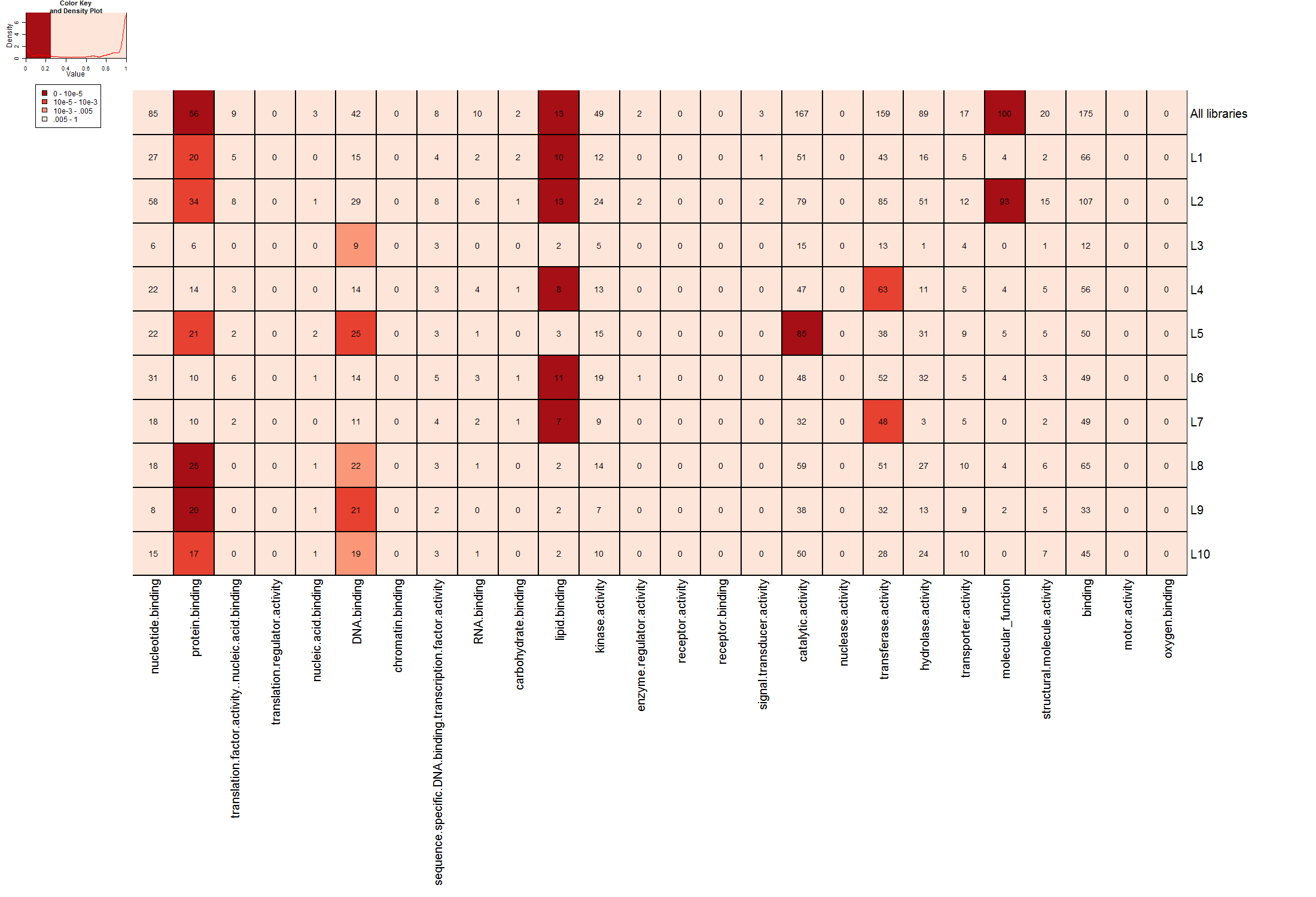** |
| --- |

| **b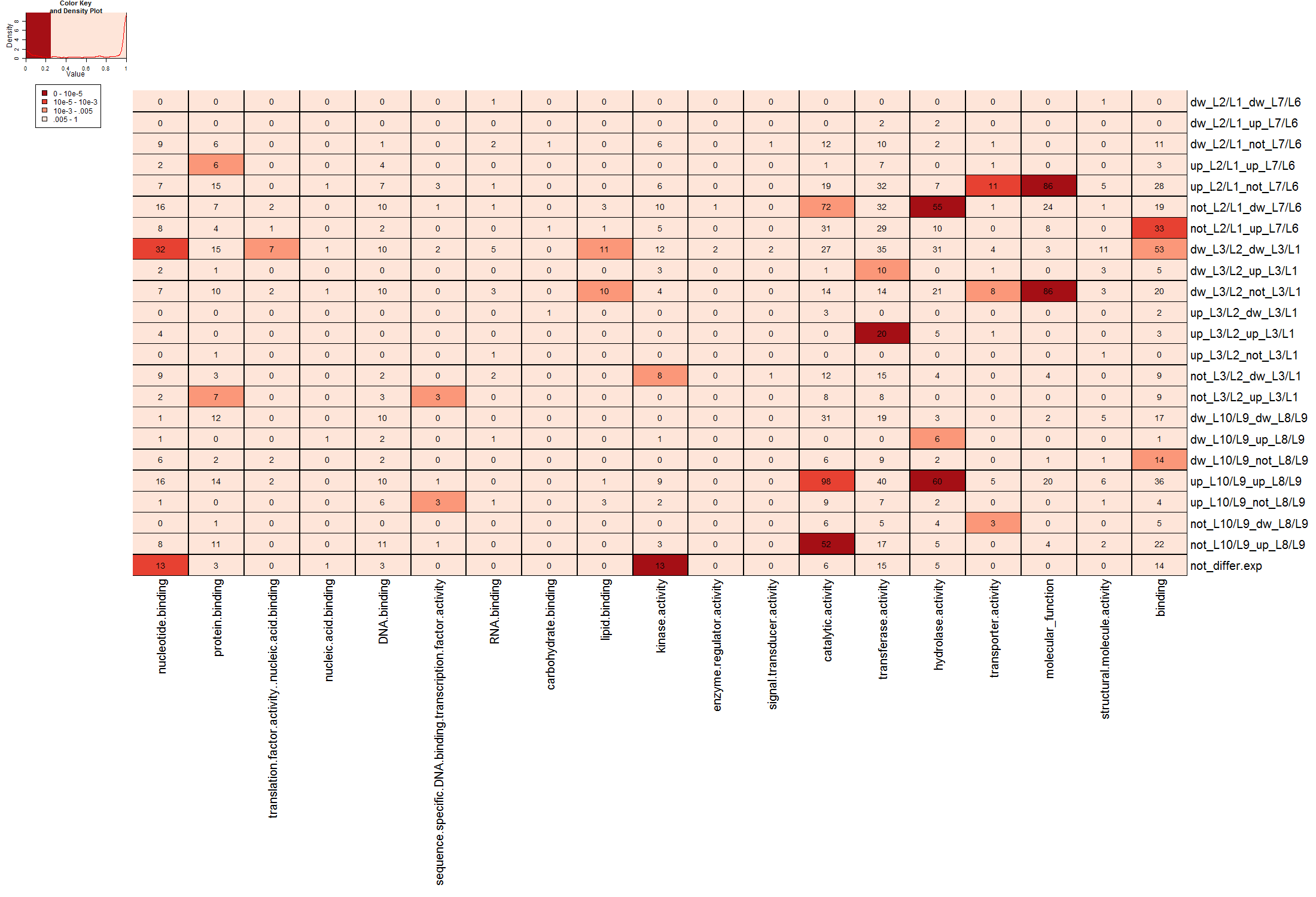** |
| --- |

**Figure S5: Enrichment of main Molecular Function GO Slims.** a) Enrichment for the target genes in different libraries. b) Enrichment for the target genes of regulated miRNA candidates, under cold, Al, and development, grouped on 24 groups (eight per investigated condition) based on their differential expression patterns described in Additional file 2: Table S11. Dw,down regulated; up, up-regulated; not, not regulated. The value in each case indicates the number of miRNA-GO associations for the corresponding GO Slim.Targets associated with more than one cell component were assigned to more than one GO Slim term.The enrichment is presented in four different colors (high enrichment (P-value < 10^-5^), medium enrichment (P-value < 10^-3^), low enrichment (P-value < 0.05) and no enrichment (P-value ≥ 0.05). MiRNA groups having targets not annotated with a UniRef are not presented.

| **a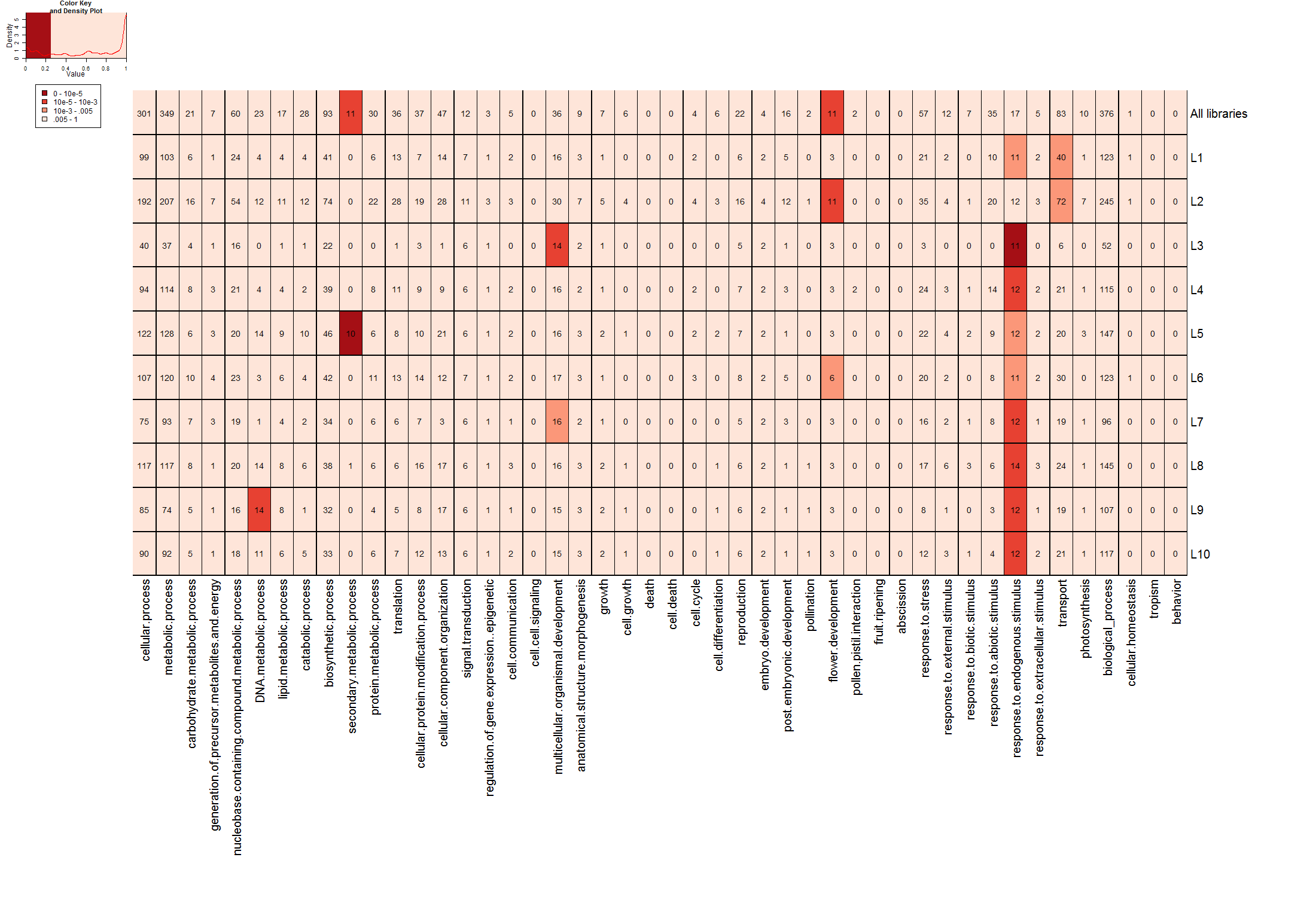** |
| --- |

| **b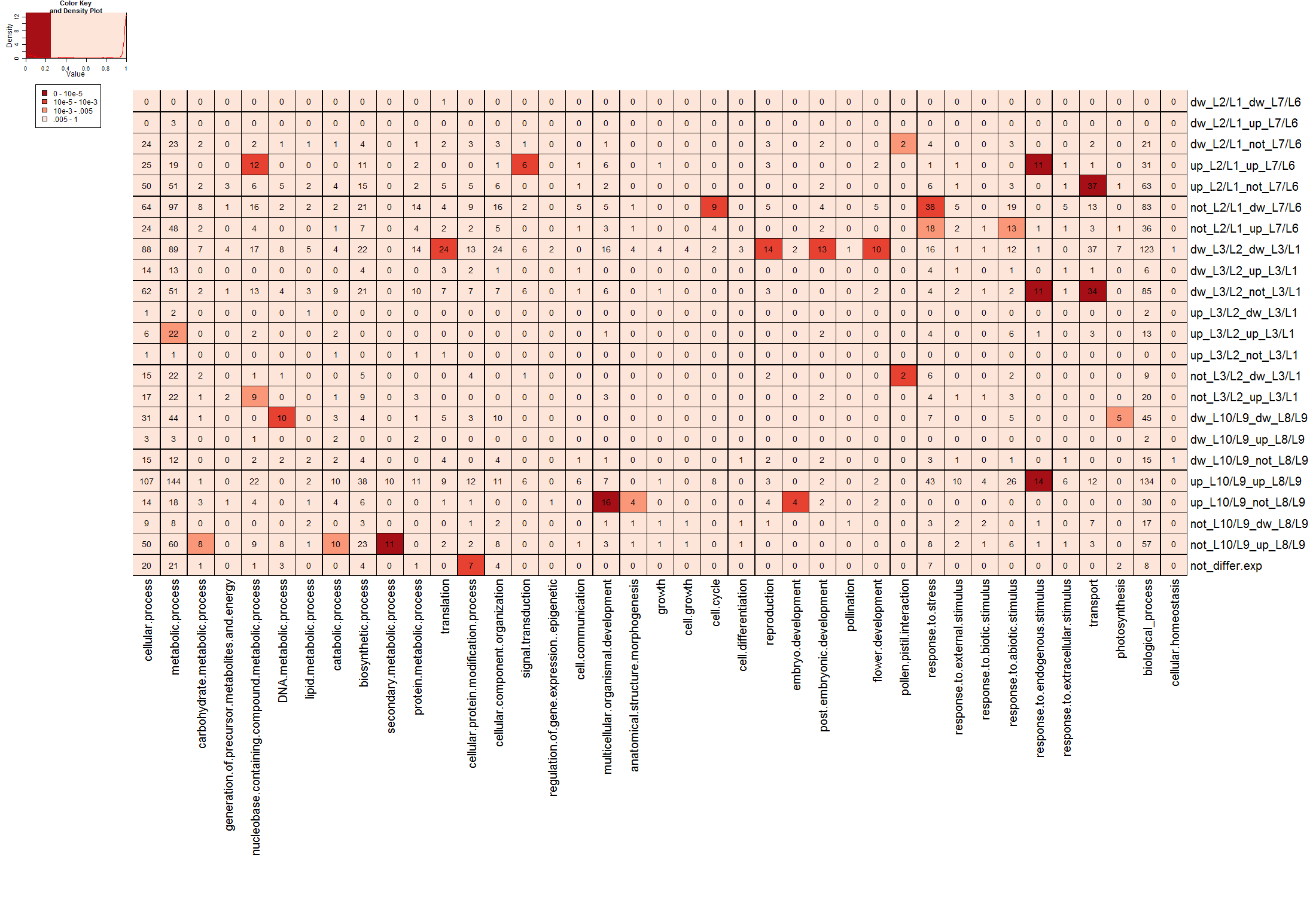** |
| --- |

**Figure S6: Enrichment of main Biological Process GO Slims.** a) Enrichment for the target genes in different libraries. b) Enrichment for the target genes of regulated miRNA candidates, under cold, Al, and development, grouped on 24 groups (eight per investigated condition) based on their differential expression patterns described in Additional file S2: Table S11. Dw,down regulated; up, up-regulated; not, not regulated. The value in each case indicates the number of miRNA-GO associations for the corresponding GO Slim.Targets associated with more than one cell component were assigned to more than one GO Slim term.The enrichment is presented in four different colors (high enrichment (P-value < 10^-5^), medium enrichment (P-value < 10^-3^), low enrichment (P-value < 0.05) and no enrichment (P-value ≥ 0.05). MiRNA groups having targets not annotated with a UniRef are not presented.


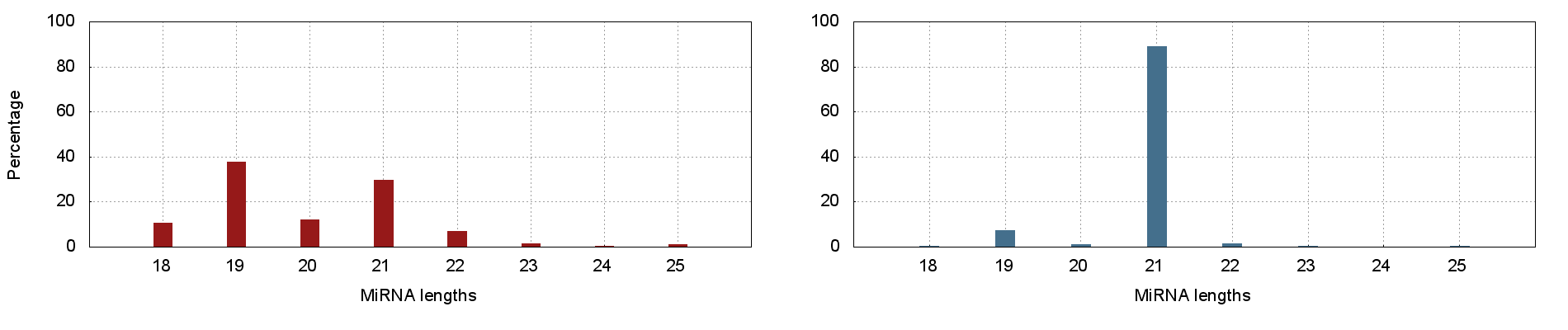

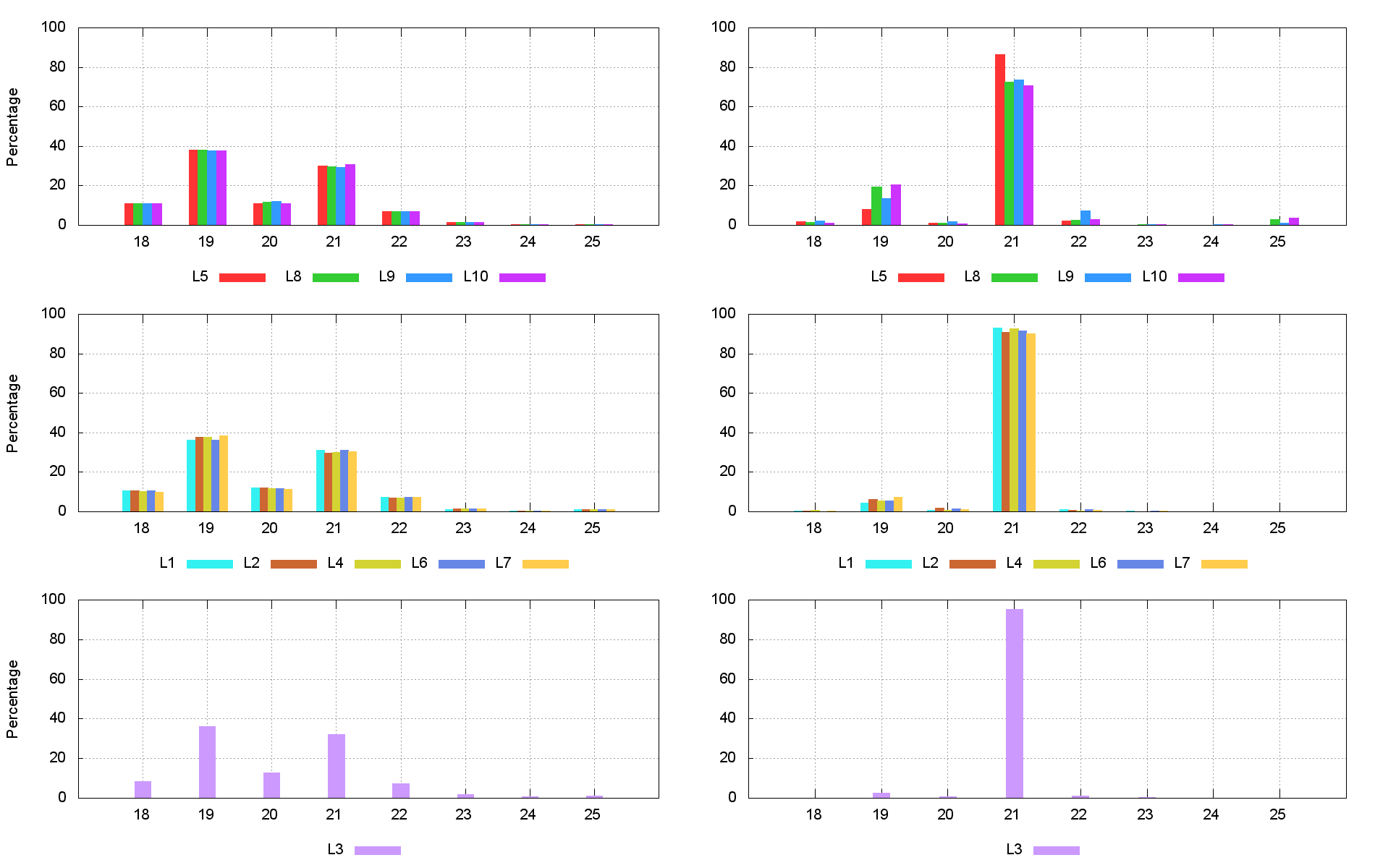

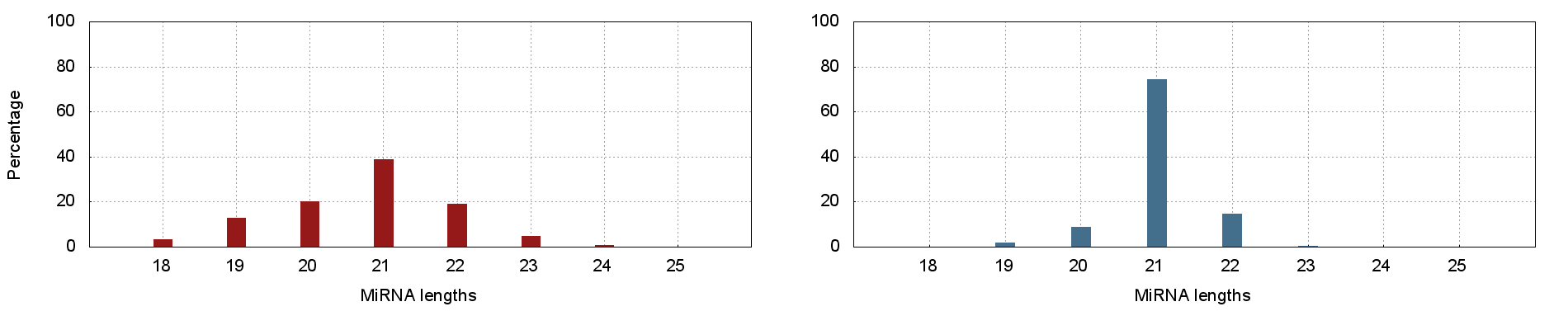


**Conserved miRNAs**

**Redundantss**

**Uniques**

**Roots**

**Reproductive tissues**

**Aerial parts**

**All tissues**

**dd**

**cc**

**ee**

**b**

**aaa**

# Figure S7: miRNA length distribution in different tissues of hexaploid wheat. Percentages of unique (different sequences) or total redundant sequences were plotted according to the length of miRNAs. New miRNAs identified from a) all tissues (aerial parts, roots and reproductive tissues) from the ten libraries; b) roots (L5, L8, L9 and L10); c) aerial parts (L1, L2, L4, L6, L7); d) reproductive tissues (L3) independently of stress and genotypes; e) conserved miRNAs in the ten sequenced libraries identified using homology searches against miRBase. For detailed information about the libraries and conditions see Additional file 1: Method S1 and Additional file 2: Table S1*.*
