# Supplementary material for: An integrative approach to identify hexaploid wheat miRNAome associated with development and tolerance to abiotic stress
Source: BMC Genomics. 2015 Apr 24;16(1):339. doi: 10.1186/s12864-015-1490-8 (PMC4443513; doi:10.1186/s12864-015-1490-8)
Supplement: Additional file 4: — Supporting Data from S1 to S3; Provides supporting Data of the manuscript and their associated legends. Their legends are in the main supplementary files named. < Supporting Data_legendeSD1toSD3.docx>. The three datasets present an elegant representation of the miRNA within their coexpressed small RNAs, their percentage of coverage and the list of miRNA with more than 100 reads. [file 12864_2015_1490_MOESM4_ESM.zip › Additional file 4/Supporting Data_legendeSD1toSD3.docx]

**Additional file 4. All the supplementary Data included in this study from Data SD1 to Data SD3**

**Data SD1**. The attached file ***SD1_viewMicroRNAinPremicroRNA.txt*** exhibits the miRNA in the context of their folded pre-miRNA in association with all the small RNAs expressed in the given context. The predictors (HHMMiR or MiPred), the dataset training used by MiRdup* for the prediction of miRNA position in the precursor and the obtained score for each dataset are presented for each precursor. Line starts with (#): Precursor sequence. Lines start with (&): Secondary structure of the precursor(s) in dot-bracket notation; Precursor id (apPre_xxxx); accesssion number of EST(s) producing the precursor(s), {Uniref ID if available}. Lines start with (>) : MiRNA sequence; Expression level of the miRNA in the 10 libraries corresponding to the number of reads sequenced in library L1 to L10; [total expression of miRNA candidates]; apMir xxxx, miRNA apMir ID; md, MiRdup*; mdB, mdP and mdM represents dataset training used by MiRdup* to predict the position of miRNA candidate in the precursor: mdB, md trained on miRBase (B); mdP, md trained on plants (P); mdM, md trained on monocots (M); mdB, mdP or mdM _Precursor ID_MiRdup score, the dataset training_ the Id of the precursor(s) from which the miRNA is predicted_the prediction score of MiRdup; HHMMiR, and/or MiPred, the predictors used to predict the secondary structure; +/-, the strand. Lines start with (%) : small RNA (sRNA) sequence mapping the precursor; Expression level of the sRNA in the 10 libraries corresponding to the number of reads sequenced in library L1 to L10; [total expression of small RNAs]; MiRNA apMir ID if this sRNA is predicted by HHMMiR or MiPred; Precursor apPre ID if this sRNA is predicted with the current precursor with HHMMiR or MiPred; +/-, strand. consmRNA_xxxx {miRxxx}, apMir having sequence homology (0-2 mismatches) with a given miRNA family from miRase(v21)**.** This data is presented in annexes.

# Data SD2.The attached file*SD2_MicroRNAsAbundancesSmall.txt*exhibits sorted miRNAs according to their percentage of overall abundance of small RNAs covering the hairpins. They are divided in three groups: higher than 50% ([100..51]), between 50% and 30% [50..31], below 30% [30..10]. #, pre-miRNA ID; >, miRNA sequence mapped in a given pre-miRNA; %, small RNA sequence mapped in a given pre-miRNA; ( ), represents the number of reads in each sequenced library; {}, represents the total of reads in the 10 sequenced libraries. This data is presented in annexes. For detailed information about the libraries and conditions see Additional file 1: Method S1 and Additional file 2: Table S1.

**Data SD3.**The attached file ***SD3_MicroRNAstotalAbundancesInAllSequencedLibraries.txt*** exhibits the miRNAs classified based on their total abundance in the ten libraries.
