# Supplementary material for: An integrative approach to identify hexaploid wheat miRNAome associated with development and tolerance to abiotic stress
Source: BMC Genomics. 2015 Apr 24;16(1):339. doi: 10.1186/s12864-015-1490-8 (PMC4443513; doi:10.1186/s12864-015-1490-8)
Supplement: Additional file 5: — Appendix-GO enrichment all targets.pdf. Gene ontology enrichment for the predicted target genes; Description of the data: Summary of all the enriched Go terms. [file 12864_2015_1490_MOESM5_ESM.pdf]

**Additional file 5: Gene ontology enrichment for predicted target genes:**

GO enrichment analysis of predicted miRNAs targets in separate and all combined libraries revealed that they may localize in diverse cellular compartments, play various functions in diverse biological and physiological processes. Targets related to the *cell component* category are associated with 21 GO Slim terms from which the term nucleus (P-value = 9.1e-004) shows enrichment in the development library (L3) suggesting their possible implication in regulating gene expression during reproductive phase. Target genes related to the *molecular function* category are represented by 19 GO Slim terms from which six show significant enrichment (Additional file 2: Table S7 and Additional file 3: Figure S5a). The most enriched terms for all the libraries is *lipid binding activity* (P-value = 7.6e-006) and *protein binding activity* (P-value = 1.2e-006). They include specific regulatory proteins and cell metabolism enzymes. Hence, the miRNA candidates are mainly associated with the modulation of several transcription factors, histones proteins and cellular enzymes involved in lipid metabolism and oxidative stress. For the targets from the roots libraries (L5 and L8 to L10), a significant enrichment is found for *protein binding activity* (P-value ranger from 2.4e-004 to 5.3e-007) and *DNA binding activity* (P-value ranger from 1.0e-003 to 1.0e-005) suggesting their possible implication in regulation of gene expression during roots development. Targets related to the *biological process* category are classified into 39 GO Slim terms, out of which six show enrichments. Targets from all the ten libraries are enriched for *secondary metabolic process* (P-value = 3.7e-005) whose related targets are known to function in both normal and stress conditions. The libraries L1 and L3 to L10 exhibited significant enrichment for response to endogenous stimulus overrepresented by auxin responsive proteins (P-value ranger from 7.8e-002 to 4.4e-007) (Additional file 2: Table S7 and Additional file 3: Figure S6a). Consistent with the investigated conditions, the targets from vernalized library (L2) corresponding to the floral transition shows a significant enrichment for *flower development* (P-value = 2.9e-005) and those from the reproductive library (L3) are enriched for *multicellular organismal development* (P-value = 1.7e-005). These include several genes with various functions in stress responses and meristem initiation, regulation of flower development, root morphogenesis and leaf development (Additional file 2: Table S7 and Figure S6a). These results support the possible function of the identified miRNAs in stress response and plant development.
